# Supplementary material for: Associations between women’s empowerment and child development, growth, and nurturing care practices in sub-Saharan Africa: A cross-sectional analysis of demographic and health survey data
Source: PLoS Med. 2021 Sep 16;18(9):e1003781. doi: 10.1371/journal.pmed.1003781 (PMC8483356; doi:10.1371/journal.pmed.1003781)
Supplement: S1 Text — (DOCX) [file pmed.1003781.s002.docx]

**S1 Text. Definition and measurement of women’s empowerment**

No standardized definition of women’s empowerment exists in the literature [1–3]. Typically, women’s empowerment is defined as the process of gaining more gender equity or changing from a state of gender inequity to a state of gender equity [1,2]. Most often women’s empowerment is defined as “the processes by which those who have been denied the ability to make choices acquire such an ability” [4].

Women’s empowerment is multi-dimensional [4–6]. Multiple and varying dimensions of women’s empowerment have been proposed in the literature primarily stemming from Kabeer’s model which distinguishes three inter-related dimensions of empowerment: resources (pre-conditions), agency (processes) and achievements (outcomes) [4]. Resources represent the pre-conditions (i.e., material, human and social resources, and institutional environments) which “enhance the ability to exercise choice” [4], i.e., the pre-conditions through which agency can be achieved. As a measure of potential for agency, resources determine the conditions in which women make choices, not the choices themselves [4]. Agency, or “the ability to define one’s goals and act upon them”, can take the form of decision-making, bargaining and negotiation, as well as other tangible and intangible processes [4]. Agency is most often measured using indicators for mobility, community and political participation, male violence, decision-making [4], and control over reproductive and sexual decisions [7]. Thus, agency itself is a multi-dimensional construct [7]. Finally, achievements are defined as the realizations of one’s goals, and may include educational, labour, or health achievements [4]. Although empowerment dimensions are often described and labelled differently in the literature (e.g., psychological, social, economic, legal, political [2,8]), most represent resources and agency as defined by Kabeer.

This multidimensionality of women’s empowerment presents a particular measurement challenge [2]. Women’s empowerment indicators are aggregated and disaggregated in different ways making comparability across studies and findings problematic [2,7]. Although women may have high empowerment in some dimensions, but not others, the use of dichotomized indicators for women’s empowerment is particularly problematic [8]. Dichotomized indicators cannot be directly compared [8] and can lead to conflicting associations with women’s and children’s outcomes [2,9]. With few exceptions, most studies construct and compare measures of women’s empowerment across countries without conducting cross-country measurement validation. Thus, in most cases it remains unclear whether women’s empowerment operates in the same way in different countries and whether cross-country comparisons of the association between women’s empowerment and women’s and children’s outcomes is appropriate [7]. Finally, some studies provide no or limited conceptual and theoretical grounding in the women’s empowerment literature, make ad hoc use of available data, and do not test the psychometric properties of their proposed empowerment measures [7,8,10].

**References**

1. Santoso M V, Kerr RB, Hoddinott J, Garigipati P, Olmos S, Young SL. Role of Women’s Empowerment in Child Nutrition Outcomes: A Systematic Review. Adv Nutr. 2019. doi:10.1093/advances/nmz056

2. Pratley P. Associations between quantitative measures of women’s empowerment and access to care and health status for mothers and their children: A systematic review of evidence from the developing world. Soc Sci Med. 2016;169: 119–131. doi:10.1016/j.socscimed.2016.08.001

3. Doku DT, Bhutta ZA, Neupane S. Associations of women’s empowerment with neonatal, infant and under-5 mortality in low- and /middle-income countries: meta-analysis of individual participant data from 59 countries. BMJ Glob Heal. 2020;5: e001558. doi:10.1136/bmjgh-2019-001558

4. Kabeer N. Resources, Agency, Achievements: Reflections on the Measurement of Women’s Empowerment. Dev Change. 1999;30: 435–464. doi:10.1111/1467-7660.00125

5. Malhotra A, Schuler SR. Women’s empowerment as a variable in international development. In: Narayan D, editor. Measuring empowerment: Cross-disciplinary perspectives. Washington: The World Bank; 2005. pp. 71–88.

6. Agarwala R, Lynch SM. Refining the Measurement of Women’s Autonomy: An International Application of a Multi-dimensional Construct. Soc Forces. 2006;84: 2077–2098. doi:10.1353/sof.2006.0079

7. Miedema SS, Haardörfer R, Girard AW, Yount KM. Women’s empowerment in East Africa: Development of a cross-country comparable measure. World Dev. 2018;110: 453–464. doi:10.1016/j.worlddev.2018.05.031

8. Richardson RA. Measuring Women’s Empowerment: A Critical Review of Current Practices and Recommendations for Researchers. Soc Indic Res. 2018;137: 539–557. doi:10.1007/s11205-017-1622-4

9. Carlson GJ, Kordas K, Murray-Kolb LE. Associations between women’s autonomy and child nutritional status: A review of the literature. Matern Child Nutr. 2015;11: 452–482. doi:10.1111/mcn.12113

10. Yount KM, Peterman A, Cheong YF. Measuring women’s empowerment: a need for context and caution. Lancet Glob Heal. 2018;6: e29. doi:10.1016/S2214-109X(17)30459-X
