# Supplementary material for: Associations between women’s empowerment and child development, growth, and nurturing care practices in sub-Saharan Africa: A cross-sectional analysis of demographic and health survey data
Source: PLoS Med. 2021 Sep 16;18(9):e1003781. doi: 10.1371/journal.pmed.1003781 (PMC8483356; doi:10.1371/journal.pmed.1003781)
Supplement: S1 Appendix — Table A. Coding of indicators from Demographic and Health Survey (DHS) variables used to describe the dimensions and subdimensions of women’s empowerment. Table B. Indicators of women’s empowerment excluded from the exploratory factor analysis and reasons for exclusion. Table C. Proportion of women endorsing each indicator of women’s empowerment. (DOCX) [file pmed.1003781.s003.docx]

**S1 Appendix. Methods to derive women’s empowerment factor scores**

We operationalized women’s empowerment as a multi-dimensional latent construct with four latent dimensions: (1) access to and control over resources, “Resources” for brevity, (2) decision-making, (3) social resources, and (4) gender attitudes, “Gender” for brevity. **Table A** summarizes the coding of DHS variables considered to describe each dimension of women’s empowerment. Since the DHS do not collect data on social resources, we proceeded with a three-dimensional model of empowerment. The “Resources” dimension was examined using four indicators for access to resources (i.e., women’s employment, type of earnings, work seasonality, and income relative to partner), three indicators for control over resources (i.e., decision-making with respect to own and partner’s income, access to money for healthcare) and two indictors for mobility (i.e., permission to seek healthcare, and ability to go alone). The “Decision-making” dimension was examined using three indicators for household decision-making (i.e., decision-making with respect to own healthcare, large household purchases, and family visits), and six indicators for reproductive health decision-making (i.e., contraception use, contraception non-use, pregnancy wanted, desire for children, ability to say no to sex, and ability to ask partner to use condom). Finally, the “Gender” dimension was examined using five indicators for attitudes towards domestic violence and was therefore renamed to “Attitudes towards wife-beating”.

All indicators were coded as binary with 1 representing empowerment, and 0 representing lack of empowerment, except for indicators for woman’s occupation, work seasonality, and income relative to partner. The latter were coded as categorical variables with higher values indicating higher empowerment. “Don’t know” responses were coded as missing. All decision-making indicators were coded as 1 if the woman decided alone or jointly with her husband/partner, and 0 otherwise. However, since it is unclear whether joint decision-making represents disguised male decision-making or cooperation [1], we conducted sensitivity analysis recoding decision-making indicators as 1 if the woman decided alone, and 0 otherwise.

To ensure cross-country comparability, we restricted indicators to those available in all countries [2,3] and asked of all married women (**Table B**). The proportions of women endorsing each indicator are shown in **Table C**.

The final list of women’s empowerment indicators (Table 2) was used to perform exploratory factor analysis (EFA) to evaluate the dimensionality of the women’s empowerment construct. We performed EFA using one random split-half sample for the pooled sample and one random split-half sample for each country. For this analysis, we used all women with available data on women’s empowerment indicators. We used a mean- and variance-adjusted weighted least squares (WLSMV) estimator, which is suitable for the binary and ordinal indicators we have, oblique rotation (GEOMIN) to allow factors to be correlated and to estimate factor correlations [4], and country-specific sampling weights provided with the data. EFA was conducted in Mplus Version 8.4 [4].

EFA on the pooled data showed five factors with eigenvalues >1 (**Figure A in S2 Appendix**), and we therefore ran five sequential models with one, two, three, four and five factors to test the fit of alternative solutions. Model fit was assessed using the following goodness of fit indices and acceptability thresholds: Comparative Fit Index (CFI) ≥ 0.95, Root Mean Square Error of Approximation (RMSEA) ≤ 0.08, and Standardized Root Mean Squared Residual (SRMR) ≤ 0.08 [5]. Since χ^2^ fit statistics and SRMR are sample size dependent, whereas CFI is relatively unaffected by sample size [6] and RMSEA is unaffected by model complexity [7], greater evaluative emphasis was placed on CFI and RMSEA. Factor correlations were used to evaluate discriminant validity with correlations ≥0.8 indicating poor discriminant validity and suggesting a more parsimonious solution [8]. The final model was selected based on model fit indices, discriminant validity and theoretical justification. Individual indicators were assessed in the pooled model and separately in each country model. Indicators with low communalities (i.e., absolute factor loadings <0.3) and indicators with cross-loadings (i.e., absolute factor loadings >0.3 on more than one factor) were dropped [8], unless dropping them led to factors with only one or two indicators [6] or maintaining them was theoretically justified.

We used confirmatory factor analysis (CFA) on one random split-half sample to test the best-fitting model from the EFA. We used a WLSMV estimator and accounted for clustering and representativeness by applying country-specific cluster variables and sampling weights. Model fit was assessed using CFI, RMSEA and SRMR as described above. Based on the CFA results in the pooled sample, the occupation indicator was excluded from the “Resources” dimension since it was nearly perfectly correlated with the remainder of indicators in this dimension and the model estimated a negative variance. A three-factor CFA model was fit for Burundi and Rwanda, based on the EFA results for these countries. No other model modifications were made. CFA was conducted in Mplus Version 8.4 [4].

To address issues of cross-country comparability of women’s empowerment [9–11], we tested for measurement invariance across countries [12,13]. First, we applied EFA and CFA separately in one random split-half sample for each country to explore the factor structure and confirm the final measurement model. Second, we used multi-group CFA to test for measurement invariance across countries by sequentially constraining factor loadings, means and residual variances. More constrained models, indicating higher measurement invariance, were retained if ΔCFI ≤ 0.01 [7]. Multi-group CFA was conducted on a reduced model, which included only indicators common across all countries [14], i.e., the “access to healthcare” dimension which did not emerge in Burundi and Rwanda was excluded. The “decision on women’s income use” was dropped from the multi-group CFA since it produced nearly perfect correlations with “income relative to partner” in two of the countries.

**References**

1. Kabeer N. Conflicts Over Credit: Re-Evaluating the Empowerment Potential of Loans to Women in Rural Bangladesh. World Dev. 2001;29: 63–84. doi:10.1016/S0305-750X(00)00081-4

2. Desai S, Johnson K. Women’s decision-mkaing and child health: familial and social hierarchies. In: Kishor S, editor. A Focus on Gender Collected Papers on Gender Using DHS Data. Calverton, Maryland, USA: ORC Macro; 2005. pp. 55–68.

3. Ewerling F, Lynch JW, Victora CG, van Eerdewijk A, Tyszler M, Barros AJD. The SWPER index for women’s empowerment in Africa: development and validation of an index based on survey data. Lancet Glob Heal. 2017;5: e916–e923. doi:10.1016/S2214-109X(17)30292-9

4. Muthén L., Muthén BO. Mplus User’s Guide. Eighth Edition. Los Angeles, CA: Muthén & Muthén; 2017.

5. Hu L, Bentler PM. Cutoff criteria for fit indexes in covariance structure analysis: Conventional criteria versus new alternatives. Struct Equ Model A Multidiscip J. 1999;6: 1–55. doi:10.1080/10705519909540118

6. Kline RB. Principles and practice of structural equation modeling. 4 edition. The Guilford Press; 2015.

7. Cheung GW, Rensvold RB. Evaluating Goodness-of-Fit Indexes for Testing Measurement Invariance. Struct Equ Model A Multidiscip J. 2002;9: 233–255. doi:10.1207/S15328007SEM0902_5

8. Brown TA. The Common Factor Model and Exploratory Factor Analysis. First. Confirmatory Factor Analysis for Applied Research. First. New York: The Guilford Press; 2006. pp. 12–39.

9. Santoso M V, Kerr RB, Hoddinott J, Garigipati P, Olmos S, Young SL. Role of Women’s Empowerment in Child Nutrition Outcomes: A Systematic Review. Adv Nutr. 2019. doi:10.1093/advances/nmz056

10. Pratley P. Associations between quantitative measures of women’s empowerment and access to care and health status for mothers and their children: A systematic review of evidence from the developing world. Soc Sci Med. 2016;169: 119–131. doi:10.1016/j.socscimed.2016.08.001

11. Carlson GJ, Kordas K, Murray-Kolb LE. Associations between women’s autonomy and child nutritional status: A review of the literature. Matern Child Nutr. 2015;11: 452–482. doi:10.1111/mcn.12113

12. Agarwala R, Lynch SM. Refining the Measurement of Women’s Autonomy: An International Application of a Multi-dimensional Construct. Soc Forces. 2006;84: 2077–2098. doi:10.1353/sof.2006.0079

13. Miedema SS, Haardörfer R, Girard AW, Yount KM. Women’s empowerment in East Africa: Development of a cross-country comparable measure. World Dev. 2018;110: 453–464. doi:10.1016/j.worlddev.2018.05.031

14. Brown TA. Confirmatory Factor Analysis for Applied Research. Second. New York: The Guilford Press; 2006.

**Table A** Coding of indicators from Demographic and Health Survey (DHS) variables used to describe the dimensions and sub-dimensions of women’s empowerment.

| **Dimension** | **Sub-dimension** | **Indicator label** | **DHS Question (variable)** | **DHS response categories^a^** | **Recode used in analysis** |
| --- | --- | --- | --- | --- | --- |
| **Access to and**  **control over**  **resources** | Access to resources | Occupation | Have you done any work in the last 12 months? (v731) | 0 = No  1 = In the past year  2 = Currently working  3 = Have a job, but on leave last 7 days | 0 = if Q=0  1 = if Q=1  2 = if Q=2 or Q=3 |
|  |  | Earnings | Are you paid in cash or kind for this work or are you not paid at all? (v741) | 0 = Not paid  1 = Cash only  2 = Cash and in-kind  3 = In-kind only | 0 = if Q=0 or Not working  1 = if Q=1 or Q=2 or Q=3 |
|  |  | Seasonality | Do you usually work throughout the year, or do you work seasonally, or only once in a while? (v732) | 1 = All year  2 = Seasonal  3 = Occasional | 0 = if Q=Not working  1 = if Q=2 or 3  2 = if Q=1 |
|  |  | Income relative to partner | Would you say that the money that you earn is more than what your (husband/partner) earns, less than what he earns, or about the same? (v746) | 1 = More than him  2 = Less than him  3 = About the same  4 = Husband/partner has no earnings  8 = Don’t know | 0 = if woman does not earn cash  1 = if Q=4  2 = if Q=2  3 = if Q=3  4 = if Q=1 |
|  | Control over income | Decision on women’s income use | Who usually decides how the money you earn will be used? (v739) | 1 = Respondent alone  2 = Respondent and husband/partner  3 = Respondent and other person  4 = Husband/partner alone  5 = Someone else  6 = Other | 0 = if woman does not earn income  0 = if Q=3 or Q=4 or Q=5 or Q=6  1 = if Q=1 or Q=2 |
|  |  | Decision on partner's income use | Who usually decides how your (husband’s/partner’s) earnings will be used? (v743f) | 1 = Respondent alone  2 = Respondent and husband/partner  3 = Respondent and other person  4 = Husband/partner alone  5 = Someone else  6 = Other  7 = Husband/partner has no earnings | 0 = if Q=3 or Q=4 or Q=5 or Q=6 or Q=7  1 = if Q=1 or Q=2 |
|  |  | Access to healthcare: Money | When you are sick and want to get medical advice or treatment, is each of the following a big problem or not a big problem: getting money needed for advice or treatment? (v467c) | 0 = No problem  1 = Big problem  2 = Not a big problem | 0 = if Q=1  1 = if Q=0 or Q=2 |
|  | Mobility | Access to healthcare: Permission to go | When you are sick and want to get medical advice or treatment, is each of the following a big problem or not a big problem: getting permission to go to the doctor? (v467b) |  |  |
|  |  | Access to healthcare: Going alone | When you are sick and want to get medical advice or treatment, is each of the following a big problem or not a big problem: not wanting to go alone? (v467f) |  |  |
| **Decision-making** | Household decision-making | Decision on own health care | Who usually makes decisions about health care for yourself? (v743a) | 1 = Respondent alone  2 = Respondent and husband/partner  3 = Respondent and other person  4 = Husband/partner alone  5 = Someone else  6 = Other | 0 = if Q=3 or Q=4 or Q=5 or Q=6  1 = if Q=1 or Q=2 |
|  |  | Decision on large household  purchases | Who usually makes decisions about major household purchases? (v743b) |  |  |
|  |  | Decision on family visits | Who usually makes decisions about visits to your family or relatives? (v743d) |  |  |
|  | Reproductive health decision-making | Contraception use | Would you say that using contraception is mainly your decision, mainly your (husband's/partner's) decision, or did you both decide together? (v632) | 1 = Mainly respondent  2 = Mainly husband/partner  3 = Joint decision  6 = Other | 0 = if Q=2 or Q=6  1 = if Q=1 or Q=3 |
|  |  | Contraception non-use | Would you say that not using contraception is mainly your decision, mainly your (husband's/partner's) decision, or did you both decide together? (v632a) |  |  |
|  |  | Pregnancy wanted | When you got pregnant, did you want to get pregnant at that time? (v225) | 1 = Then  2 = Later  3 = Not at all | 0 = if Q=2 or Q=3  1 = if Q=1 |
|  |  | Desire for children | Does your (husband/partner) want the same number of children that you want, or does he want more or fewer than you want? (v621) | 1 = Both want same  2 = Husband wants more  3 = Husband wants fewer  8 = Don’t know | 0 = if Q=2 or Q=3 or Q=8  1 = if Q=1 |
|  |  | Can say no to sex | Can you say no to your (husband/partner) if you do not want to have sexual intercourse? (v850a) | 0 = No  1 = Yes  8 = Don’t know/not sure/depends | 0 = if Q=0 or Q=8  1 = if Q=1 |
|  |  | Can ask partner to use condom | Could you ask your (husband/partner) to use a condom if you wanted him to? (v850b) |  |  |
| **Attitudes towards wife-beating** | Wife beating | Goes out without telling husband | In your opinion, is a husband justified in hitting or beating his wife if she goes out without telling him? (v744a) | 0 = No  1 = Yes  8 = Don’t know | 0 = if Q=1  1 = if Q=0 |
|  |  | Neglects children | In your opinion, is a husband justified in hitting or beating his wife if she neglects the children? (v744b) |  |  |
|  |  | Argues with husband | In your opinion, is a husband justified in hitting or beating his wife if she argues with him? (v744c) |  |  |
|  |  | Refuses sex | In your opinion, is a husband justified in hitting or beating his wife if she refuses to have sex with him? (v744d) |  |  |
|  |  | Burns food | In your opinion, is a husband justified in hitting or beating his wife if she burns the food? (v744e) |  |  |

^a^ “Don’t know” responses were coded as missing.

**Table B** Indicators of women’s empowerment excluded from the exploratory factor analysis and reasons for exclusion

| **Dimension** | **Indicator label** | **Excluded** | **Reason for exclusion** |
| --- | --- | --- | --- |
| **Access to and control over resources** | Occupation | No |  |
|  | Earnings | Yes | Perfectly correlated with “Income relative to partner” |
|  | Seasonality | No |  |
|  | Income relative to partner | No |  |
|  | Decision on women’s income use | No |  |
|  | Decision on partner's income use | No |  |
|  | Access to healthcare: Money | No |  |
|  | Access to healthcare: Permission to go | No |  |
|  | Access to healthcare: Going alone | No |  |
| **Decision-making** | Decision on own health care | No |  |
|  | Decision on large household  purchases | No |  |
|  | Decision on family visits | No |  |
|  | Contraception use | Yes | Only asked to women using contraception |
|  | Contraception non-use | Yes | Only asked to women using contraception |
|  | Pregnancy wanted | Yes | Only asked of currently pregnant women |
|  | Desire for children | Yes | Only asked to women who do not use female or male sterilization as a contraception method |
|  | Can say no to sex | No |  |
|  | Can ask partner to use condom | No |  |
| **Attitudes towards wife-beating** | Wife beating: Goes out without telling husband | No |  |
|  | Wife beating: Neglects children | No |  |
|  | Wife beating: Argues with husband | Yes | Not available in all surveys (Congo) |
|  | Wife beating: Refuses sex | No |  |
|  | Wife beating: Burns food | Yes | Not available in all surveys (Congo) |

**Table C** Proportion of women endorsing each indicator of women’s empowerment^a^

|  | **Pooled sample (N=86,743)** | **Benin (N=11,170)** | **Burundi (N=9,559)** | **Cameroon (N=9,804)** | **Chad (N=13,437)** | **Congo (N=6,750)** | **Rwanda (N=6,890)** | **Senegal (N=11,394)** | **Togo (N=6,360)** | **Uganda (N=11,379)** |
| --- | --- | --- | --- | --- | --- | --- | --- | --- | --- | --- |
| Worked in last 12 months: No | 23.55 | 15.62 | 6.50 | 24.48 | 47.23 | 23.75 | 5.77 | 37.27 | 14.99 | 16.21 |
| Worked in last 12 months: In the past year | 5.22 | 2.04 | 5.85 | 5.32 | 8.92 | 2.30 | 7.51 | 7.92 | 2.78 | 3.90 |
| Worked in last 12 months: Currently working | 71.23 | 82.34 | 87.64 | 70.20 | 43.85 | 73.95 | 86.72 | 54.81 | 82.23 | 79.90 |
| Worked all year: Not working | 23.57 | 15.62 | 6.50 | 24.51 | 47.34 | 23.76 | 5.77 | 37.27 | 15.01 | 16.21 |
| Worked all year: Seasonal, occasional work | 29.50 | 26.79 | 18.21 | 34.13 | 38.55 | 20.92 | 28.18 | 26.31 | 22.85 | 31.64 |
| Worked all year: Yes | 46.93 | 57.59 | 75.28 | 41.36 | 14.11 | 55.31 | 66.05 | 36.42 | 62.14 | 52.15 |
| Income relative to partner: Does not earn cash | 41.93 | 29.42 | 48.16 | 34.76 | 64.72 | 30.16 | 27.48 | 49.70 | 31.06 | 35.14 |
| Income relative to partner: Partner does not earn | 0.78 | 0.83 | 0.57 | 0.66 | 1.03 | 0.16 | 1.47 | 1.01 | 0.36 | 0.78 |
| Income relative to partner: Less than him | 46.61 | 58.45 | 38.67 | 55.39 | 30.76 | 57.11 | 47.19 | 43.69 | 58.37 | 49.13 |
| Income relative to partner: About the same | 6.08 | 5.74 | 8.19 | 3.78 | 1.47 | 7.33 | 16.90 | 2.99 | 4.46 | 8.67 |
| Income relative to partner: More than him | 4.59 | 5.55 | 4.40 | 5.41 | 2.02 | 5.24 | 6.96 | 2.62 | 5.76 | 6.29 |
| Decision on women’s income use | 53.64 | 66.38 | 45.59 | 61.74 | 32.63 | 61.76 | 63.53 | 47.98 | 68.57 | 59.49 |
| Decision on partner’s income use | 37.64 | 26.08 | 64.02 | 39.07 | 17.08 | 47.83 | 74.95 | 19.82 | 16.57 | 49.04 |
| Access to healthcare: Money for doctor | 42.35 | 46.88 | 37.35 | 25.37 | 23.00 | 35.02 | 50.69 | 52.40 | 38.77 | 56.33 |
| Access to healthcare: Permission to go to the doctor | 83.33 | 77.41 | 94.62 | 69.02 | 55.27 | 53.50 | 97.83 | 93.41 | 86.98 | 94.80 |
| Access to healthcare: Not wanting to go alone to health centre | 78.70 | 81.79 | 83.74 | 76.00 | 55.37 | 69.63 | 84.20 | 86.20 | 85.50 | 78.80 |
| Decision on own health care | 48.12 | 46.36 | 72.04 | 38.22 | 25.02 | 39.82 | 83.83 | 26.31 | 42.10 | 73.60 |
| Decision on large household purchases | 50.72 | 47.17 | 69.30 | 47.82 | 40.03 | 59.88 | 73.58 | 21.01 | 47.27 | 63.74 |
| Decision on family visits | 59.86 | 66.53 | 81.25 | 53.62 | 47.40 | 62.09 | 85.69 | 35.40 | 64.70 | 71.94 |
| Can say no to sex | 61.32 | 56.89 | 60.19 | 74.67 | 44.67 | 70.73 | 82.65 | 17.79 | 71.89 | 85.57 |
| Can ask partner to use a condom | 54.69 | 40.14 | 59.11 | 57.33 | 17.39 | 65.57 | 83.31 | 24.26 | 60.23 | 79.00 |
| Wife beating justified if she goes out without telling husband | 63.81 | 76.45 | 61.53 | 70.81 | 36.33 | 58.38 | 79.43 | 60.75 | 80.02 | 69.63 |
| Wife beating justified if she neglects children | 59.14 | 75.22 | 49.48 | 62.17 | 36.31 | 52.86 | 72.20 | 59.69 | 79.18 | 61.58 |
| Wife beating justified if she refuses to have sex with husband | 71.02 | 85.69 | 57.69 | 82.86 | 54.56 | 71.33 | 76.50 | 57.19 | 88.95 | 81.41 |

^a^ All values are percentages unless indicated otherwise. Country statistics account for survey design using sampling weights.
