# Supplementary material for: Associations between women’s empowerment and child development, growth, and nurturing care practices in sub-Saharan Africa: A cross-sectional analysis of demographic and health survey data
Source: PLoS Med. 2021 Sep 16;18(9):e1003781. doi: 10.1371/journal.pmed.1003781 (PMC8483356; doi:10.1371/journal.pmed.1003781)
Supplement: S2 Appendix — Fig A. Scree plot from exploratory factor analysis conducted on one random split-half sample. Table A. Exploratory factor analysis of women’s empowerment dimensions on one random split-half sample. (DOCX) [file pmed.1003781.s004.docx]

**S2 Appendix. Exploratory factor analysis (EFA) results**

The **Figure A** below shows the EFA scree plot, and the **Table A** shows the EFA results from the three-, four-, and five-factor solutions in the pooled sample. A five-factor model showed the most adequate fit: χ^2^ (50) = 6914.47, p < 0.001, CFI = 0.994, RMSEA = 0.056, and SRMR = 0.041. However, the five-factor model yielded a two-indicator factor (reproductive health decision). Therefore, a more parsimonious four-factor model was selected as the final model despite RMSEA and SRMR not attaining the *a priori* defined thresholds: χ^2^ (62) = 31913.74, p < 0.001, CFI = 0.985, RMSEA = 0.084, and SRMR = 0.08. In this four-factor model, no indicators were dropped due to low communalities or cross-loadings. Four of the five indicators on “Resources” loaded on the first factor with loadings ranging from 0.862 to 1.026. The fifth indicator related to decision-making on partner’s income loaded on the second factor together with indicators related to “Decision-making”. Loadings ranged from 0.698 to 0.912. Loadings for the third factor, described by indicators on “Access to healthcare”, ranged from 0.634 to 0.879. Finally, indicators on attitudes towards wife-beating had large loadings (0.800 to 0.944) on the fourth factor “Attitudes towards wife-beating”.

These findings were supported across all countries. The four-factor model showed adequate fit: CFI ranged from 0.973 (Rwanda) to 0.997 (Cameroon and Congo), RMSEA ranged from 0.039 (Chad and Congo) to 0.096 (Togo), and SRMR ranged from 0.049 (Congo) to 0.088 (Togo). The two indicators related to reproductive health were excluded due to low communalities (Burundi, Chad, Congo, Rwanda, and Togo) or loading on a single two-item factor (Cameroon, Senegal, and Uganda). Although these indicators had acceptable loadings in Benin, they were dropped to maintain comparability across countries. No indicators were excluded due to cross-loadings in any of the countries.

Similar to the results from the pooled sample, four of the five indicators on “Resources” loaded on the first (Benin, Chad, Congo, Senegal, Togo, Uganda) or second (Rwanda) factor, whereas the fifth indicator related to decision-making on partner’s income loaded on the second (Senegal, Uganda) or third (Benin, Burundi, Chad, Congo, Rwanda, Togo) factor together with indicators related to “Decision-making”. In contrast, indicators on “Resources” loaded on two separate factors in Burundi. Loadings for indicators on “Resources” ranged from 0.529 (Rwanda) to 1.043 (Congo), and those for “Decision-making” ranged from 0.470 (Congo) to 0.991 (Chad). Lastly, the three indicators related to attitude towards wife-beating loaded on a single factor with loadings ranging from 0.685 (Chad) to 0.976 (Senegal).

Although we hypothesized that “Access to healthcare” was part of the “Resources” dimension of women’s empowerment, “Access to healthcare” indicators loaded on a different factor in the pooled sample and separately in all countries except for Burundi, Cameroon, Rwanda, and Uganda. Loadings ranged from 0.267 (Senegal) to 0.939 (Congo). The “Access to healthcare” factor was identified in Cameroon and Uganda after re-estimating the four-factor EFA model excluding the reproductive health indicators with low communalities. However, a three-factor model without an “Access to healthcare” factor remained optimal in Burundi and Rwanda.

In the pooled sample, factors showed acceptable discriminant validity. Factor correlations were all positive and significant, ranging from 0.048 to 0.407. Across countries, discriminant validity was also acceptable though some factor correlations were negative or not statistically significant.

**Figure A** Scree plot from exploratory factor analysis conducted on one random split-half sample


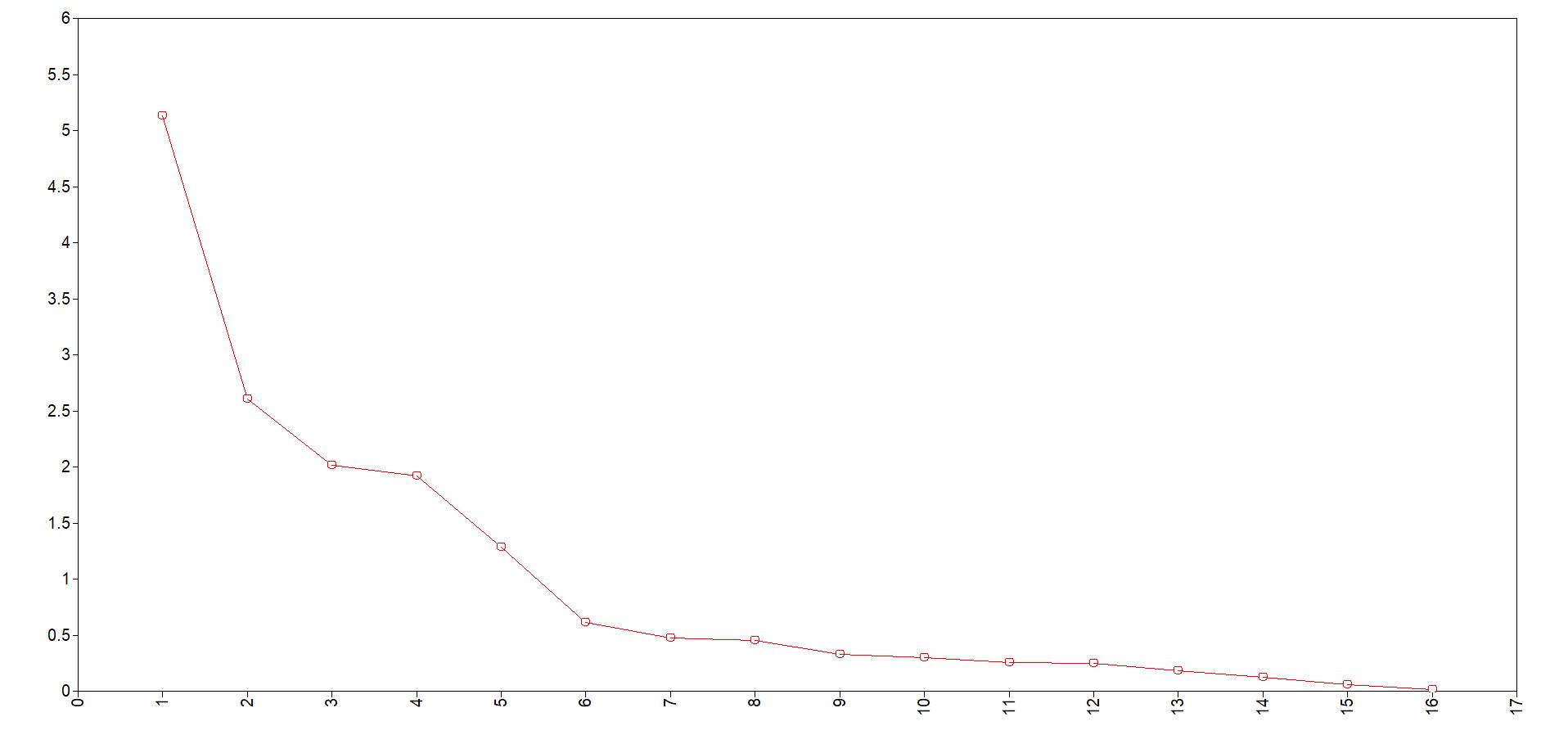


**Table A** Exploratory factor analysis of women’s empowerment dimensions on one random split-half sample^a^

| **3-factor solution**^b^ | | | |  | **4-factor solution**^c^ | | | |  | **5-factor solution**^d^ | | | | |
| --- | --- | --- | --- | --- | --- | --- | --- | --- | --- | --- | --- | --- | --- | --- |
|  | F1 | F2 | F3 |  | F1 | F2 | F3 | F4 |  | F1 | F2 | F3 | F4 | F5 |
| Occupation | 0.905 | 0.31 | -0.028 |  | 1.016 | 0.042 | -0.467 | -0.010 |  | 0.949 | -0.035 | 0.001 | -0.071 | 0.547 |
| Seasonality of earnings | 0.799 | 0.281 | 0.021 |  | 0.862 | 0.056 | -0.350 | 0.033 |  | 0.803 | -0.021 | 0.050 | -0.012 | 0.462 |
| Income relative to partner | 0.888 | 0.005 | 0.288 |  | 0.945 | -0.081 | 0.036 | -0.021 |  | 0.865 | 0.160 | -0.035 | 0.051 | -0.008 |
| Decision on women’s income use | 0.984 | -0.008 | 0.354 |  | 1.026 | -0.097 | 0.066 | 0.012 |  | 0.932 | 0.190 | -0.015 | 0.089 | -0.053 |
| Decision on partner's income use | -0.008 | 0.708 | -0.054 |  | 0.016 | 0.704 | 0.037 | -0.070 |  | -0.019 | 0.698 | 0.049 | -0.058 | 0.044 |
| Money not a problem to access healthcare | -0.063 | 0.109 | 0.160 |  | -0.044 | 0.092 | 0.600 | 0.028 |  | -0.031 | -0.065 | 0.718 | 0.005 | -0.001 |
| Getting permission to go not a problem to access healthcare | -0.037 | 0.267 | 0.194 |  | 0.008 | 0.240 | 0.709 | 0.014 |  | 0.004 | 0.062 | 0.879 | -0.022 | 0.035 |
| Not wanting to go alone not a problem to access healthcare | -0.010 | 0.133 | 0.220 |  | 0.027 | 0.094 | 0.591 | 0.074 |  | 0.031 | 0.010 | 0.634 | 0.072 | -0.064 |
| Decision on own health care | 0.036 | 0.886 | 0.004 |  | 0.072 | 0.871 | 0.033 | -0.025 |  | 0.029 | 0.883 | 0.022 | 0.004 | 0.030 |
| Decision on large household purchases | 0.019 | 0.857 | -0.044 |  | 0.047 | 0.853 | -0.002 | -0.068 |  | 0.001 | 0.912 | -0.065 | -0.031 | -0.021 |
| Decision on family visits | 0.018 | 0.724 | 0.074 |  | 0.046 | 0.717 | 0.057 | 0.047 |  | 0.007 | 0.731 | 0.043 | 0.071 | 0.015 |
| Can say no to sex | -0.048 | 0.508 | 0.094 |  | -0.041 | 0.514 | -0.269 | 0.184 |  | -0.005 | 0.076 | -0.089 | 0.102 | 0.736 |
| Can ask partner to use a condom | -0.068 | 0.533 | 0.084 |  | -0.058 | 0.536 | -0.196 | 0.160 |  | -0.027 | 0.093 | 0.021 | 0.067 | 0.721 |
| Wife beating justified: Goes out without telling husband | 0.010 | 0.017 | 0.925 |  | 0.022 | 0.016 | 0.020 | 0.922 |  | 0.007 | 0.024 | 0.021 | 0.921 | 0.007 |
| Wife beating justified: Neglects children | 0.000 | -0.067 | 0.937 |  | 0.009 | -0.068 | 0.020 | 0.939 |  | -0.003 | -0.039 | 0.004 | 0.944 | -0.035 |
| Wife beating justified: Refuses sex | -0.001 | 0.046 | 0.791 |  | 0.008 | 0.048 | -0.008 | 0.801 |  | -0.001 | 0.002 | -0.007 | 0.800 | 0.099 |
| *EFA fit statistics* |  |  |  |  |  |  |  |  |  |  |  |  |  |  |
| χ^2^ (p-value) | 31913.737 (p<0.0001) | | |  | 18982.808 (p<0.0001) | | | |  | 6914.474 (p<0.0001) | | | | |
| CFI | 0.974 |  |  |  | 0.985 |  |  |  |  | 0.994 |  |  |  |  |
| RMSEA | 0.099 |  |  |  | 0.084 |  |  |  |  | 0.056 |  |  |  |  |
| SRMR | 0.109 |  |  |  | 0.080 |  |  |  |  | 0.041 |  |  |  |  |
| *Factor correlations* |  |  |  |  |  |  |  |  |  |  |  |  |  |  |
| F1 | 1.000 |  |  |  | 1.000 |  |  |  |  | 1.000 |  |  |  |  |
| F2 | 0.240 | 1.000 |  |  | 0.407 | 1.000 |  |  |  | 0.248 | 1.000 |  |  |  |
| F3 | -0.090 | 0.217 | 1.000 |  | 0.187 | 0.048 | 1.000 |  |  | 0.053 | 0.168 | 1.000 |  |  |
| F4 |  |  |  |  | 0.134 | 0.200 | 0.120 | 1.000 |  | 0.063 | 0.188 | 0.143 | 1.000 |  |
| F5 |  |  |  |  |  |  |  |  |  | -0.057 | 0.379 | 0.115 | 0.112 | 1.000 |

^a^ EFA, exploratory factor analysis; CFI, Comparative Fit Index; RMSEA, Root Mean Square Error of Approximation; SRMR, Standardized Root Mean Squared Residual

^b^ F1, Access to and control over resources; F2, Decision-making; F3, Attitudes towards wife-beating.

^c^ F1, Access to and control over resources; F2, Decision-making; F3, Access to healthcare; F4, Attitudes towards wife-beating.

^d^ F1, Access to and control over resources; F2, Decision-making; F3, Decision-making; F4, Attitudes towards wife-beating; F5, Reproductive health decisions.
