# Supplementary material for: Associations between women’s empowerment and child development, growth, and nurturing care practices in sub-Saharan Africa: A cross-sectional analysis of demographic and health survey data
Source: PLoS Med. 2021 Sep 16;18(9):e1003781. doi: 10.1371/journal.pmed.1003781 (PMC8483356; doi:10.1371/journal.pmed.1003781)
Supplement: S3 Appendix — Table A. Confirmatory factor analysis of women’s empowerment dimensions on one random split-half sample by country. (DOCX) [file pmed.1003781.s005.docx]

**S3 Appendix. Confirmatory factor analysis (CFA) results**

The four-factor CFA models in both the pooled sample and separately in each country (except for Burundi and Rwanda where we fit a three-factor model) showed acceptable model fit, reaching the *a priori* defined thresholds (**Table A**). CFI ranged from 0.985 (Cameroon) to 0.998 (Burundi), RMSEA ranged from 0.018 (Burundi) to 0.033 (Benin), and SRMR ranged from 0.060 (Benin) to 0.026 (Burundi). In the pooled sample, factor correlations were positive and significant, and indicated acceptable discriminant validity (all factor correlations ≤0.80). The strongest factor correlation was between “Decision-making” and “Resources” (0.444), while the weakest correlation was between “Resources” and “Access to healthcare” (0.114). By country, factor correlations varied, and some negative correlations were also observed. Analogous to the pooled sample, the strongest factor correlations were between “Decision-making” and “Resources” (ranging from 0.539 in Chad to 0.296 in Rwanda). Negative factor correlations were observed between “Resources” and “Access to healthcare” in Benin (-0.018), and between “Resources” and “Attitudes towards wife-beating” in Cameroon, Chad, and Congo (-0.014, -0.142 and -0.026 respectively).

**Table A** Confirmatory factor analysis of women’s empowerment dimensions on one random split-half sample by country^a^

|  | **Benin** | | | | **Burundi** | | | | | **Cameroon** | | | | | **Chad** | | | | | **Congo** | | | | |  |
| --- | --- | --- | --- | --- | --- | --- | --- | --- | --- | --- | --- | --- | --- | --- | --- | --- | --- | --- | --- | --- | --- | --- | --- | --- | --- |
| *Indicator* | F1 | F2 | F3 | F4 | | F1 | F2 | F3 | F4 | | F1 | F2 | F3 | F4 | | F1 | F2 | F3 | F4 | | F1 | F2 | F3 | F4 | |
| Seasonality of earnings | … |  |  |  | | … |  |  |  | | … |  |  |  | | … |  |  |  | | … |  |  |  | |
| Income relative to partner | 1.197* |  |  |  | | 5.272* |  |  |  | | 1.128* |  |  |  | | 1.083* |  |  |  | | 1.095* |  |  |  | |
| Decision on women’s income use | 1.315* |  |  |  | | 9.407* |  |  |  | | 1.260* |  |  |  | | 1.107* |  |  |  | | 1.130* |  |  |  | |
| Money not a problem to access healthcare |  | … |  |  | |  | - |  |  | |  | … |  |  | |  | … |  |  | |  | … |  |  | |
| Getting permission to go not a problem to access healthcare |  | 1.298* |  |  | |  | - |  |  | |  | 1.433* |  |  | |  | 1.368* |  |  | |  | 1.145* |  |  | |
| Not wanting to go alone not a problem to access healthcare |  | 1.005* |  |  | |  | - |  |  | |  | 0.920* |  |  | |  | 0.909* |  |  | |  | 0.782* |  |  | |
| Decision on partner's income use |  |  | … |  | |  |  | … |  | |  |  | … |  | |  |  | … |  | |  |  | … |  | |
| Decision on own health care |  |  | 1.590* |  | |  |  | 1.156* |  | |  |  | 1.778* |  | |  |  | 1.213* |  | |  |  | 1.244* |  | |
| Decision on large household purchases |  |  | 1.593* |  | |  |  | 1.233* |  | |  |  | 1.852* |  | |  |  | 1.139* |  | |  |  | 1.291* |  | |
| Decision on family visits |  |  | 1.252* |  | |  |  | 1.168* |  | |  |  | 1.730* |  | |  |  | 0.756* |  | |  |  | 0.727* |  | |
| Wife beating justified: Goes out without telling husband |  |  |  | … | |  |  |  | … | |  |  |  | … | |  |  |  | … | |  |  |  | … | |
| Wife beating justified: Neglects children |  |  |  | 0.974* | |  |  |  | 1.050* | |  |  |  | 0.973* | |  |  |  | 0.986* | |  |  |  | 0.901* | |
| Wife beating justified: Refuses sex |  |  |  | 0.884* | |  |  |  | 0.907* | |  |  |  | 0.890* | |  |  |  | 0.741* | |  |  |  | 0.878* | |
| *CFA fit statistics* |  |  |  |  | |  | | | | |  | | | | |  | | | | |  | | | | |
| χ^2^ (p-value) | 418.748 (<0.0001) | |  |  | | 79.283 (<0.0001) | |  |  | | 257.209 (<0.0001) | |  |  | | 236.171 (<0.0001) | |  |  | | 181.848 (<0.0001) | |  |  | |
| CFI | 0.993 |  |  |  | | 0.998 |  |  |  | | 0.985 |  |  |  | | 0.995 |  |  |  | | 0.991 |  |  |  | |
| RMSEA | 0.033 |  |  |  | | 0.018 |  |  |  | | 0.026 |  |  |  | | 0.021 |  |  |  | | 0.025 |  |  |  | |
| SRMR | 0.060 |  |  |  | | 0.026 |  |  |  | | 0.044 |  |  |  | | 0.048 |  |  |  | | 0.054 |  |  |  | |

|  | **Rwanda** | | | | **Senegal** | | | | **Togo** | | | | **Uganda** | | | |
| --- | --- | --- | --- | --- | --- | --- | --- | --- | --- | --- | --- | --- | --- | --- | --- | --- |
| *Indicator* | F1 | F2 | F3 | F4 | F1 | F2 | F3 | F4 | F1 | F2 | F3 | F4 | F1 | F2 | F3 | F4 |
| Seasonality of earnings | … |  |  |  | … |  |  |  | … |  |  |  | … |  |  |  |
| Income relative to partner | 1.742* |  |  |  | 1.056* |  |  |  | 1.121* |  |  |  | 1.351* |  |  |  |
| Decision on women’s income use | 2.653* |  |  |  | 1.107* |  |  |  | 1.255* |  |  |  | 1.581* |  |  |  |
| Money not a problem to access healthcare |  | - |  |  |  | … |  |  |  | … |  |  |  | … |  |  |
| Getting permission to go not a problem to access healthcare |  | - |  |  |  | 1.684* |  |  |  | 1.484* |  |  |  | 1.266* |  |  |
| Not wanting to go alone not a problem to access healthcare |  | - |  |  |  | 1.663* |  |  |  | 1.240* |  |  |  | 1.030* |  |  |
| Decision on partner's income use |  |  | … |  |  |  | … |  |  |  | … |  |  |  | … |  |
| Decision on own health care |  |  | 1.244* |  |  |  | 1.291* |  |  |  | 1.807* |  |  |  | 1.612* |  |
| Decision on large household purchases |  |  | 1.411* |  |  |  | 1.208* |  |  |  | 1.801* |  |  |  | 1.785* |  |
| Decision on family visits |  |  | 1.263* |  |  |  | 1.042* |  |  |  | 1.415* |  |  |  | 1.576* |  |
| Wife beating justified: Goes out without telling husband |  |  |  | … |  |  |  | … |  |  |  | … |  |  |  | … |
| Wife beating justified: Neglects children |  |  |  | 0.971* |  |  |  | 1.000* |  |  |  | 1.058* |  |  |  | 0.981* |
| Wife beating justified: Refuses sex |  |  |  | 0.806* |  |  |  | 0.973* |  |  |  | 0.954* |  |  |  | 0.799* |
| *CFA fit statistics* |  | | | |  | | | |  | | | |  | | | |
| χ^2^ (p-value) | 135.083 (<0.0001) | |  |  | 361.450 (<0.0001) | |  |  | 244.887 (<0.0001) | |  |  | 247.866 (<0.0001) | |  |  |
| CFI | 0.992 |  |  |  | 0.994 |  |  |  | 0.995 |  |  |  | 0.994 |  |  |  |
| RMSEA | 0.031 |  |  |  | 0.030 |  |  |  | 0.031 |  |  |  | 0.024 |  |  |  |
| SRMR | 0.041 |  |  |  | 0.054 |  |  |  | 0.057 |  |  |  | 0.042 |  |  |  |

^a^ F1, Access to and control over resources; F2, Access to healthcare; F3, Decision-making; F4, Attitudes towards wife-beating; CFA, confirmatory factor analysis; CFI, Comparative Fit Index; RMSEA, Root Mean Square Error of Approximation; SRMR, Standardized Root Mean Squared Residual
