# Supplementary material for: Associations between women’s empowerment and child development, growth, and nurturing care practices in sub-Saharan Africa: A cross-sectional analysis of demographic and health survey data
Source: PLoS Med. 2021 Sep 16;18(9):e1003781. doi: 10.1371/journal.pmed.1003781 (PMC8483356; doi:10.1371/journal.pmed.1003781)
Supplement: S4 Appendix — Table A. Estimated factor correlation matrices for the latent variables from the form-invariant confirmatory factor analysis model by country. (DOCX) [file pmed.1003781.s006.docx]

**S4 Appendix. Multi-group confirmatory factor analysis (CFA) results**

The three-factor form-invariant measurement model estimated using multi-group CFA indicated acceptable model fit with a CFI of 0.973, RMSEA of 0.043 and SRMR of 0.055. Residual variances of indicators related to income were freely estimated. Thus, our model showed that the indicators measured the same factors in each country, however, the indicators related to income were measured with a different degree of precision. Factor correlations by country are shown in the **Table**.

**Table A** Estimated factor correlation matrices for the latent variables from the form-invariant confirmatory factor analysis model by country^a^

|  | Benin | | |  | Burundi | | |  | Cameroon | | |  | Chad | | |  | Congo | | |
| --- | --- | --- | --- | --- | --- | --- | --- | --- | --- | --- | --- | --- | --- | --- | --- | --- | --- | --- | --- |
|  | F1 | F2 | F3 |  | F1 | F2 | F3 |  | F1 | F2 | F3 |  | F1 | F2 | F3 |  | F1 | F2 | F3 |
| F1 | 1 |  |  |  | 1 |  |  |  | 1 |  |  |  | 1 |  |  |  | 1 |  |  |
| F2 | 0.451 | 1 |  |  | 0.200 | 1 |  |  | 0.402 | 1 |  |  | 0.494 | 1 |  |  | 0.244 | 1 |  |
| F3 | 0.013 | 0.193 | 1 |  | -0.059 | 0.174 | 1 |  | -0.019 | 0.193 | 1 |  | -0.154 | 0.075 | 1 |  | -0.030 | 0.056 | 1 |

|  | Rwanda | | |  | Senegal | | |  | Togo | | |  | Uganda | | |
| --- | --- | --- | --- | --- | --- | --- | --- | --- | --- | --- | --- | --- | --- | --- | --- |
|  | F1 | F2 | F3 |  | F1 | F2 | F3 |  | F1 | F2 | F3 |  | F1 | F2 | F3 |
| F1 | 1 |  |  |  | 1 |  |  |  | 1 |  |  |  | 1 |  |  |
| F2 | 0.105 | 1 |  |  | 0.382 | 1 |  |  | 0.403 | 1 |  |  | 0.315 | 1 |  |
| F3 | 0.003 | 0.190 | 1 |  | 0.146 | 0.226 | 1 |  | 0.081 | 0.042 | 1 |  | 0.059 | 0.183 | 1 |

^a^ F1, Access to and control over resources; F2, Decision-making; F3, Attitudes towards wife-beating
