# Supplementary material for: Associations between women’s empowerment and child development, growth, and nurturing care practices in sub-Saharan Africa: A cross-sectional analysis of demographic and health survey data
Source: PLoS Med. 2021 Sep 16;18(9):e1003781. doi: 10.1371/journal.pmed.1003781 (PMC8483356; doi:10.1371/journal.pmed.1003781)
Supplement: S5 Appendix — Fig A. Distributions of the individual dimension factor scores and the total empowerment score overlayed with the standard normal distribution. Fig B. Correlation between individual dimension and total empowerment scores and the Gender Inequality Index. Table A. Biserial correlations between the continuous total empowerment and individual dimensions scores and all considered outcomes. Table B. Biserial correlations between the total empowerment and individual dimensions quintile categories and all considered outcomes. (DOCX) [file pmed.1003781.s007.docx]

**S5 Appendix. Empowerment score diagnostics**

**Figure A** Distributions of the individual dimension factor scores and the total empowerment score overlayed with the standard normal distribution


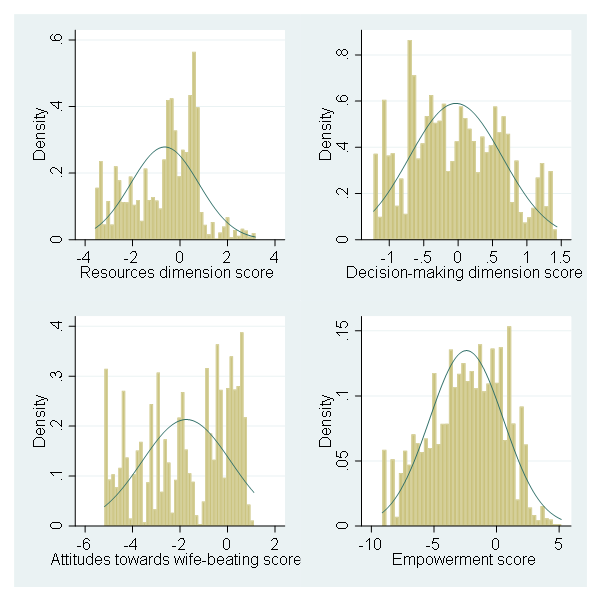


**Figure B** Correlation between individual dimension and total empowerment scores and the Gender Inequality Index


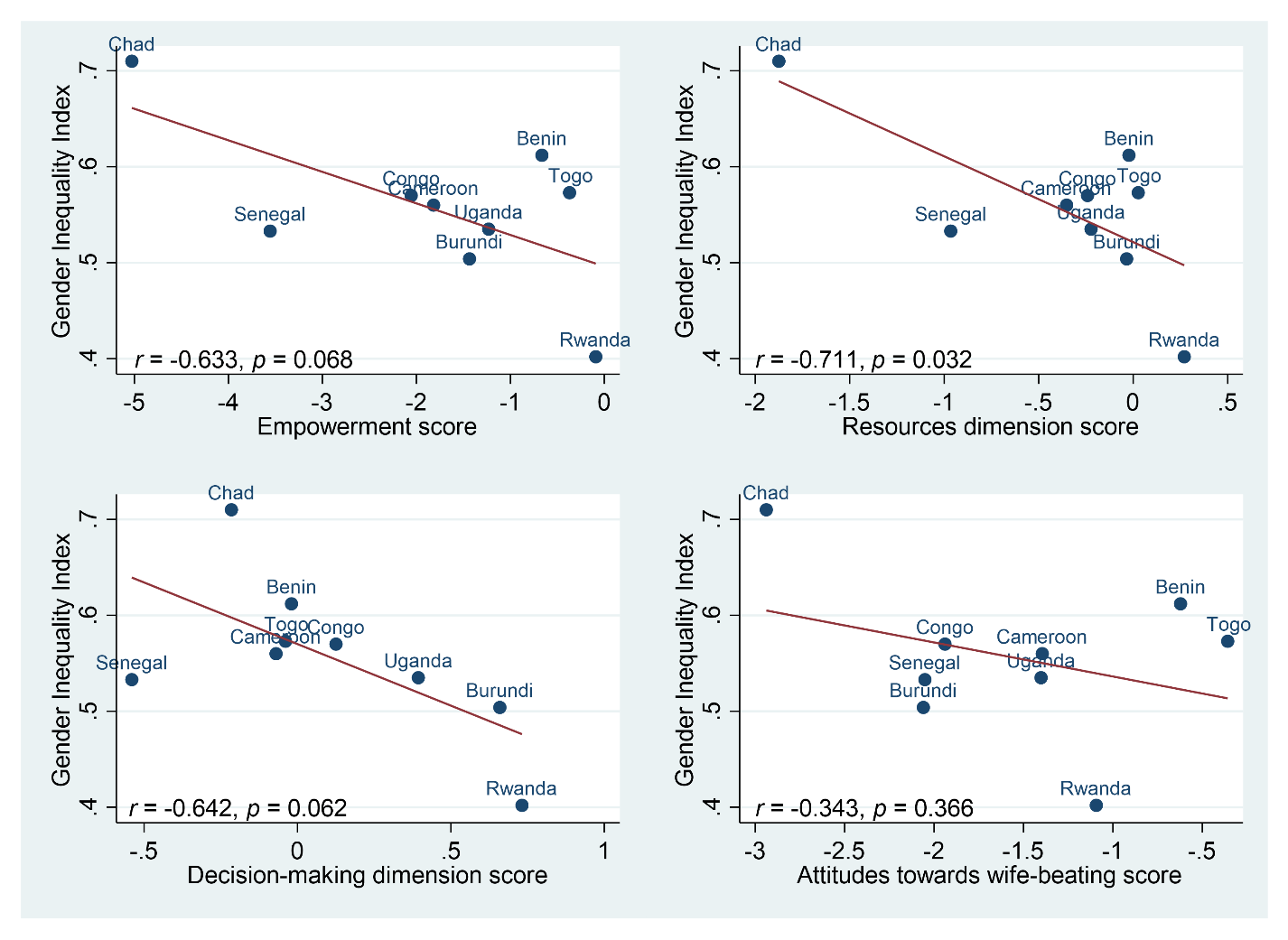


**Table A** Biserial correlations between the continuous total empowerment and individual dimensions scores and all considered outcomes^a^

|  | Empowerment score | Resources score | Decision-making score | Attitudes towards wife-beating score |
| --- | --- | --- | --- | --- |
| Cognitive development off track | -0.10** | -0.11** | -0.04** | -0.06** |
| Socio-emotional development off track | -0.03** | 0.01 | 0.01 | -0.05** |
| Literacy-numeracy development off track | -0.13** | -0.10** | -0.12** | -0.09** |
| Physical development off track | -0.07** | -0.09** | -0.07** | -0.02** |
| Overall development off track | -0.10** | -0.10** | -0.05** | -0.06** |
| HAZ | 0.06** | 0.06** | -0.05** | 0.06** |
| Stunted (HAZ <-2) | -0.05** | -0.06** | 0.06** | -0.05** |
| Number of learning resources | 0.15** | 0.10** | 0.11** | 0.13** |
| Number of maternal stimulation activities | 0.10** | 0.06** | 0.16** | 0.06** |
| ≥4 maternal stimulation activities | 0.08** | 0.06** | 0.10** | 0.04** |
| Number of paternal stimulation activities | 0.15** | 0.11** | 0.13** | 0.10** |
| ≥4 paternal stimulation activities | 0.08** | 0.07** | 0.06** | 0.06** |
| Dietary diversity score | 0.17** | 0.16** | 0.12** | 0.10** |
| Minimum dietary diversity (DDS≥4) | 0.11** | 0.09** | 0.05** | 0.09** |

^a^ Abbreviations used: HAZ, height-for-age Z-score; DDS, dietary diversity score. Statistical significance: * *p* < 0.05. ** *p* < 0.01.

**Table B** Biserial correlations between the total empowerment and individual dimensions quintile categories and all considered outcomes^a^

|  | Empowerment quintile categories | Resources quintile categories | Decision-making quintile categories | Attitudes towards wife-beating quintile categories |
| --- | --- | --- | --- | --- |
| Cognitive development off track | -0.04** | -0.06** | -0.06** | -0.03** |
| Socio-emotional development off track | -0.02* | 0.00 | -0.02* | -0.03** |
| Literacy-numeracy development off track | -0.09** | -0.07** | -0.08** | -0.07** |
| Physical development off track | -0.04** | -0.06** | -0.05** | -0.01 |
| Overall development off track | 0.07** | 0.06** | 0.05** | 0.04** |
| HAZ | -0.06** | -0.05** | -0.04** | -0.03** |
| Stunted (HAZ <-2) | -0.04** | -0.06** | -0.05** | -0.02** |
| Number of learning resources | 0.09** | 0.09** | 0.08** | 0.06** |
| Number of maternal stimulation activities | 0.07** | 0.05** | 0.07** | 0.06** |
| ≥4 maternal stimulation activities | 0.04** | 0.04** | 0.05** | 0.03** |
| Number of paternal stimulation activities | 0.08** | 0.06** | 0.07** | 0.07** |
| ≥4 paternal stimulation activities | 0.04** | 0.04** | 0.04** | 0.03** |
| Dietary diversity score | 0.05** | 0.05** | 0.05** | 0.04** |
| Minimum dietary diversity (DDS≥4) | 0.03** | 0.02** | 0.03** | 0.04** |

^a^ Abbreviations used: HAZ, height-for-age Z-score; DDS, dietary diversity score. Statistical significance: * *p* < 0.05. ** *p* < 0.01.
