# Supplementary material for: Associations between women’s empowerment and child development, growth, and nurturing care practices in sub-Saharan Africa: A cross-sectional analysis of demographic and health survey data
Source: PLoS Med. 2021 Sep 16;18(9):e1003781. doi: 10.1371/journal.pmed.1003781 (PMC8483356; doi:10.1371/journal.pmed.1003781)
Supplement: S2 Table — Table A. Associations between quintile categories s of women’s total empowerment and empowerment dimensions and early learning outcomes. Table B. Associations between women’s total empowerment and empowerment dimensions and early learning outcomes. Table C. Associations between quintile categories of women’s total empowerment and empowerment dimensions and child diet. Table D. Associations between women’s total empowerment and empowerment dimensions and child diet. (DOCX) [file pmed.1003781.s009.docx]

**S2 Table. Association of women’s empowerment and early learning and nutrition outcomes**

**Table A** Associations between quintile categories s of women’s total empowerment and empowerment dimensions and early learning outcomes^a^

|  | **Number of learning resources**  **(0-4)**  **(N=21,276)**  **MD (95% CI)** | | **Number of maternal stimulation activities (0-6)**  **(N=20,745)**  **MD (95% CI)** | | **≥4 maternal stimulation activities**  **(N=20,745)**  **RR (95% CI)** | |
| --- | --- | --- | --- | --- | --- | --- |
|  | **Unadjusted** | **Adjusted** | **Unadjusted** | **Adjusted** | **Unadjusted** | **Adjusted** |
| *Total empowerment* |  |  |  |  |  |  |
| Empowerment Q1 (lowest) | Ref. | Ref. | Ref. | Ref. | Ref. | Ref. |
| Empowerment Q2 | 0.08 (0.02, 0.13) | 0.03 (-0.03, 0.08) | 0.07 (-0.03, 0.16) | 0.07 (-0.02, 0.16) | 0.99 (0.86, 1.15) | 0.98 (0.85, 1.12) |
| Empowerment Q3 | 0.09 (0.02, 0.15) | 0.02 (-0.03, 0.08) | 0.08 (-0.02, 0.18) | 0.04 (-0.05, 0.14) | 0.95 (0.82, 1.11) | 0.90 (0.78, 1.05) |
| Empowerment Q4 | 0.14 (0.08, 0.20) | 0.04 (-0.01, 0.10) | 0.15 (0.05, 0.25) | 0.09 (0.00, 0.18) | 1.06 (0.91, 1.23) | 0.99 (0.86, 1.14) |
| Empowerment Q5 (highest) | 0.26 (0.20, 0.33) | 0.07 (0.01, 0.13) | 0.28 (0.18, 0.39) | 0.16 (0.06, 0.25) | 1.24 (1.06, 1.44) | 1.06 (0.91, 1.22) |
| *Empowerment dimensions* |  |  |  |  |  |  |
| Resources Q1 (lowest) | Ref. | Ref. | Ref. | Ref. | Ref. | Ref. |
| Resources Q2 | 0.00 (-0.06, 0.07) | 0.03 (-0.03, 0.09) | -0.11 (-0.21, -0.01) | -0.10 (-0.20, 0.00) | 0.81 (0.70, 0.95) | 0.91 (0.78, 1.05) |
| Resources Q3 | 0.02 (-0.06, 0.09) | 0.03 (-0.04, 0.10) | -0.27 (-0.38, -0.17) | -0.20 (-0.30, -0.10) | 0.69 (0.59, 0.82) | 0.78 (0.67, 0.91) |
| Resources Q4 | 0.12 (0.05, 0.19) | 0.08 (0.02, 0.14) | -0.06 (-0.17, 0.05) | -0.08 (-0.19, 0.03) | 0.93 (0.80, 1.10) | 0.96 (0.83, 1.10) |
| Resources Q5 (highest) | 0.18 (0.10, 0.26) | 0.07 (0.00, 0.15) | 0.07 (-0.05, 0.19) | 0.02 (-0.09, 0.13) | 1.02 (0.86, 1.20) | 1.03 (0.88, 1.21) |
| Decision-making Q1 (lowest) | Ref. | Ref. | Ref. | Ref. | Ref. | Ref. |
| Decision-making Q2 | 0.14 (0.07, 0.22) | 0.11 (0.03, 0.18) | 0.40 (0.27, 0.52) | 0.34 (0.22, 0.46) | 1.46 (1.21, 1.75) | 1.32 (1.10, 1.57) |
| Decision-making Q3 | 0.16 (0.09, 0.24) | 0.11 (0.04, 0.18) | 0.24 (0.13, 0.35) | 0.18 (0.08, 0.29) | 1.20 (1.00, 1.45) | 1.09 (0.92, 1.29) |
| Decision-making Q4 | 0.10 (0.01, 0.18) | 0.07 (0.00, 0.15) | 0.25 (0.13, 0.37) | 0.23 (0.12, 0.34) | 1.32 (1.09, 1.60) | 1.22 (1.02, 1.45) |
| Decision-making Q5 (highest) | 0.10 (0.01, 0.19) | 0.08 (0.00, 0.16) | 0.36 (0.22, 0.50) | 0.33 (0.20, 0.47) | 1.55 (1.25, 1.93) | 1.40 (1.14, 1.74) |
| Attitudes towards wife beating Q1 (lowest) | Ref. | Ref. | Ref. | Ref. | Ref. | Ref. |
| Attitudes towards wife beating Q2 | 0.02 (-0.04, 0.08) | -0.06 (-0.11, 0.00) | -0.14 (-0.26, -0.03) | -0.22 (-0.34, -0.11) | 0.73 (0.61, 0.88) | 0.67 (0.56, 0.79) |
| Attitudes towards wife beating Q3 | 0.10 (0.04, 0.17) | 0.02 (-0.04, 0.08) | -0.03 (-0.15, 0.08) | -0.12 (-0.23, -0.01) | 0.91 (0.76, 1.07) | 0.82 (0.70, 0.96) |
| Attitudes towards wife beating Q4 | 0.09 (0.02, 0.17) | -0.05 (-0.12, 0.02) | 0.00 (-0.12, 0.13) | -0.12 (-0.24, 0.00) | 0.92 (0.77, 1.10) | 0.78 (0.65, 0.93) |
| Attitudes towards wife beating Q5 (highest) | 0.11 (0.03, 0.19) | -0.04 (-0.11, 0.03) | 0.02 (-0.12, 0.16) | -0.12 (-0.25, 0.01) | 0.87 (0.72, 1.06) | 0.74 (0.62, 0.89) |

|  | **Number of paternal stimulation activities (0-6)**  **(N=20,745)**  **MD (95% CI)** | | **≥4 paternal stimulation activities**  **(N=20,745)**  **RR (95% CI)** | |
| --- | --- | --- | --- | --- |
|  | **Unadjusted** | **Adjusted** | **Unadjusted** | **Adjusted** |
| *Total empowerment* |  |  |  |  |
| Empowerment Q1 (lowest) | Ref. | Ref. | Ref. | Ref. |
| Empowerment Q2 | 0.07 (0.02, 0.13) | 0.08 (0.03, 0.13) | 1.24 (0.89, 1.74) | 1.20 (0.87, 1.67) |
| Empowerment Q3 | 0.08 (0.03, 0.14) | 0.09 (0.03, 0.14) | 1.18 (0.87, 1.60) | 1.14 (0.84, 1.55) |
| Empowerment Q4 | 0.12 (0.06, 0.17) | 0.11 (0.05, 0.16) | 1.28 (0.94, 1.75) | 1.22 (0.90, 1.65) |
| Empowerment Q5 (highest) | 0.26 (0.20, 0.32) | 0.23 (0.17, 0.29) | 2.06 (1.56, 2.72) | 1.79 (1.34, 2.38) |
| *Empowerment dimensions* |  |  |  |  |
| Resources Q1 (lowest) | Ref. | Ref. | Ref. | Ref. |
| Resources Q2 | 0.09 (0.03, 0.15) | 0.09 (0.04, 0.15) | 1.18 (0.81, 1.72) | 1.35 (0.94, 1.94) |
| Resources Q3 | 0.05 (-0.01, 0.12) | 0.08 (0.02, 0.14) | 1.18 (0.84, 1.67) | 1.29 (0.95, 1.75) |
| Resources Q4 | 0.09 (0.02, 0.16) | 0.09 (0.03, 0.16) | 1.25 (0.86, 1.81) | 1.28 (0.92, 1.79) |
| Resources Q5 (highest) | 0.18 (0.11, 0.26) | 0.17 (0.10, 0.24) | 1.42 (0.99, 2.04) | 1.45 (1.04, 2.01) |
| Decision-making Q1 (lowest) | Ref. | Ref. | Ref. | Ref. |
| Decision-making Q2 | 0.06 (0.01, 0.12) | 0.04 (-0.01, 0.09) | 1.05 (0.74, 1.48) | 0.92 (0.66, 1.29) |
| Decision-making Q3 | -0.02 (-0.08, 0.05) | -0.02 (-0.08, 0.04) | 0.78 (0.52, 1.16) | 0.73 (0.51, 1.06) |
| Decision-making Q4 | 0.05 (-0.03, 0.12) | 0.05 (-0.02, 0.12) | 1.19 (0.79, 1.78) | 1.14 (0.79, 1.65) |
| Decision-making Q5 (highest) | 0.02 (-0.06, 0.10) | 0.04 (-0.04, 0.11) | 1.28 (0.85, 1.92) | 1.29 (0.88, 1.91) |
| Attitudes towards wife beating Q1 (lowest) | Ref. | Ref. | Ref. | Ref. |
| Attitudes towards wife beating Q2 | 0.11 (0.05, 0.17) | 0.05 (0.00, 0.11) | 1.32 (0.95, 1.84) | 1.09 (0.80, 1.49) |
| Attitudes towards wife beating Q3 | 0.10 (0.05, 0.16) | 0.05 (0.00, 0.11) | 1.22 (0.88, 1.69) | 1.03 (0.76, 1.41) |
| Attitudes towards wife beating Q4 | 0.16 (0.10, 0.22) | 0.09 (0.03, 0.15) | 1.62 (1.18, 2.22) | 1.26 (0.93, 1.72) |
| Attitudes towards wife beating Q5 (highest) | 0.22 (0.15, 0.29) | 0.13 (0.07, 0.20) | 1.57 (1.10, 2.25) | 1.14 (0.81, 1.62) |

^a^ All estimates accounted for clustering and representativeness using the country-specific cluster variables and sampling weights. Adjusted estimates controlled for household wealth, rurality, and size; household head’s age and sex; maternal education, age, and age at first co-habitation; child age and sex; country and survey year. Abbreviations used: Q, quintile category; Ref, reference; MD, mean difference; RR, relative risk.

**Table B** Associations between women’s total empowerment and empowerment dimensions and early learning outcomes^a^

|  | **Number of learning resources**  **(0-4)**  **(N=21,276)**  **MD (95% CI)** | | **Number of maternal stimulation activities (0-6)**  **(N=20,745)**  **MD (95% CI)** | | **≥4 maternal stimulation activities**  **(N=20,745)**  **RR (95% CI)** | |
| --- | --- | --- | --- | --- | --- | --- |
|  | **Unadjusted** | **Adjusted** | **Unadjusted** | **Adjusted** | **Unadjusted** | **Adjusted** |
| Total empowerment | 0.05 (0.05, 0.06) | 0.01 (0.00, 0.02) | 0.05 (0.04, 0.06) | 0.02 (0.00, 0.03) | 1.07 (1.05, 1.09) | 1.01 (0.98, 1.03) |
| *Dimensions* |  |  |  |  |  |  |
| Resources | 0.05 (0.03, 0.07) | 0.02 (0.01, 0.04) | 0.00 (-0.03, 0.03) | 0.01 (-0.02, 0.04) | 1.06 (1.01, 1.10) | 1.01 (0.97, 1.06) |
| Decision-making | 0.06 (0.03, 0.10) | 0.03 (-0.01, 0.06) | 0.37 (0.31, 0.43) | 0.20 (0.13, 0.27) | 1.35 (1.25, 1.46) | 1.25 (1.13, 1.38) |
| Attitudes towards wife beating | 0.06 (0.04, 0.07) | 0.00 (-0.02, 0.01) | 0.00 (-0.01, 0.02) | -0.02 (-0.04, 0.00) | 1.01 (0.98, 1.03) | 0.94 (0.91, 0.98) |

|  | **Number of paternal stimulation activities (0-6)**  **(N=20,745)**  **MD (95% CI)** | | **≥4 paternal stimulation activities**  **(N=20,745)**  **RR (95% CI)** | |
| --- | --- | --- | --- | --- |
|  | **Unadjusted** | **Adjusted** | **Unadjusted** | **Adjusted** |
| Total empowerment | 0.05 (0.05, 0.06) | 0.03 (0.02, 0.04) | 1.18 (1.15, 1.22) | 1.11 (1.07, 1.16) |
| *Dimensions* |  |  |  |  |
| Resources | 0.05 (0.03, 0.06) | 0.03 (0.02, 0.05) | 1.19 (1.10, 1.29) | 1.07 (0.98, 1.16) |
| Decision-making | 0.13 (0.09, 0.16) | 0.08 (0.04, 0.12) | 1.40 (1.21, 1.61) | 1.52 (1.22, 1.91) |
| Attitudes towards wife beating | 0.04 (0.03, 0.05) | 0.02 (0.01, 0.03) | 1.12 (1.07, 1.18) | 1.04 (0.97, 1.12) |

^a^ All estimates accounted for clustering and representativeness using the country-specific cluster variables and sampling weights. Adjusted estimates controlled for household wealth, rurality, and size; household head’s age and sex; maternal education, age, and age at first co-habitation; child age and sex; country and survey year. Abbreviations used: MD, mean difference; RR, relative risk.

**Table C** Associations between quintile categories of women’s total empowerment and empowerment dimensions and child diet^a^

|  | **Dietary diversity score (DDS, 0-7)**  **(N=11,279)**  **MD (95% CI)** | | **Minimum dietary diversity (DDS≥4)**  **(N=11,279)**  **RR (95% CI)** | |
| --- | --- | --- | --- | --- |
|  | **Unadjusted** | **Adjusted** | **Unadjusted** | **Adjusted** |
| *Total empowerment* |  |  |  |  |
| Empowerment Q1 (lowest) | Ref. | Ref. | Ref. | Ref. |
| Empowerment Q2 | 0.06 (-0.06, 0.18) | 0.06 (-0.06, 0.17) | 1.09 (0.91, 1.31) | 1.07 (0.90, 1.28) |
| Empowerment Q3 | 0.09 (-0.03, 0.21) | 0.07 (-0.05, 0.18) | 1.07 (0.90, 1.27) | 1.02 (0.86, 1.20) |
| Empowerment Q4 | 0.12 (0.00, 0.24) | 0.08 (-0.03, 0.20) | 1.08 (0.90, 1.30) | 1.01 (0.85, 1.20) |
| Empowerment Q5 (highest) | 0.25 (0.13, 0.37) | 0.17 (0.06, 0.29) | 1.29 (1.09, 1.53) | 1.07 (0.91, 1.27) |
| *Empowerment dimensions* |  |  |  |  |
| Resources Q1 (lowest) | Ref. | Ref. | Ref. | Ref. |
| Resources Q2 | 0.00 (-0.13, 0.13) | 0.02 (-0.11, 0.14) | 0.90 (0.75, 1.07) | 0.97 (0.81, 1.16) |
| Resources Q3 | -0.02 (-0.16, 0.12) | 0.02 (-0.11, 0.15) | 0.85 (0.69, 1.05) | 0.89 (0.74, 1.09) |
| Resources Q4 | 0.09 (-0.05, 0.22) | 0.08 (-0.05, 0.22) | 1.04 (0.85, 1.27) | 1.01 (0.84, 1.22) |
| Resources Q5 (highest) | 0.27 (0.10, 0.44) | 0.27 (0.11, 0.44) | 1.25 (0.99, 1.58) | 1.20 (0.96, 1.49) |
| Decision-making Q1 (lowest) | Ref. | Ref. | Ref. | Ref. |
| Decision-making Q2 | 0.06 (-0.07, 0.19) | 0.08 (-0.04, 0.21) | 1.09 (0.90, 1.33) | 1.12 (0.93, 1.36) |
| Decision-making Q3 | 0.14 (0.00, 0.29) | 0.16 (0.03, 0.29) | 1.07 (0.87, 1.32) | 1.09 (0.89, 1.34) |
| Decision-making Q4 | 0.08 (-0.08, 0.24) | 0.10 (-0.05, 0.26) | 1.02 (0.81, 1.28) | 1.03 (0.82, 1.29) |
| Decision-making Q5 (highest) | -0.09 (-0.26, 0.08) | -0.04 (-0.20, 0.12) | 0.81 (0.63, 1.04) | 0.82 (0.64, 1.05) |
| Attitudes towards wife beating Q1 (lowest) | Ref. | Ref. | Ref. | Ref. |
| Attitudes towards wife beating Q2 | 0.02 (-0.10, 0.14) | -0.08 (-0.20, 0.03) | 1.07 (0.89, 1.30) | 0.93 (0.78, 1.12) |
| Attitudes towards wife beating Q3 | 0.02 (-0.10, 0.14) | -0.09 (-0.20, 0.03) | 1.17 (0.98, 1.40) | 1.02 (0.86, 1.21) |
| Attitudes towards wife beating Q4 | 0.05 (-0.06, 0.17) | -0.09 (-0.20, 0.02) | 1.14 (0.95, 1.37) | 0.91 (0.76, 1.09) |
| Attitudes towards wife beating Q5 (highest) | 0.26 (0.10, 0.41) | 0.05 (-0.09, 0.19) | 1.55 (1.28, 1.89) | 1.22 (1.00, 1.48) |

^a^ All estimates accounted for clustering and representativeness using the country-specific cluster variables and sampling weights. Adjusted estimates controlled for household wealth, rurality, and size; household head’s age and sex; maternal education, age, and age at first co-habitation; child age and sex; country and survey year. Abbreviations used: Q, quintile category; MD, mean difference; RR, relative risk; Ref, reference.

**Table D** Associations between women’s total empowerment and empowerment dimensions and child diet^a^

|  | **Dietary diversity score (DDS, 0-7)**  **(N=11,279)**  **MD (95% CI)** | | **Minimum dietary diversity (DDS≥4)**  **(N=11,279)**  **RR (95% CI)** | |
| --- | --- | --- | --- | --- |
|  | **Unadjusted** | **Adjusted** | **Unadjusted** | **Adjusted** |
| Total empowerment | 0.09 (0.08, 0.10) | 0.03 (0.01, 0.04) | 1.10 (1.08, 1.12) | 1.02 (0.99, 1.04) |
| *Dimensions* |  |  |  |  |
| Resources | 0.16 (0.12, 0.20) | 0.08 (0.05, 0.12) | 1.16 (1.09, 1.22) | 1.05 (0.99, 1.11) |
| Decision-making | 0.05 (-0.02, 0.11) | 0.01 (-0.06, 0.09) | 0.90 (0.82, 0.99) | 0.94 (0.85, 1.05) |
| Attitudes towards wife beating | 0.06 (0.03, 0.08) | 0.00 (-0.02, 0.02) | 1.12 (1.08, 1.16) | 1.02 (0.98, 1.05) |

^a^ All estimates accounted for clustering and representativeness using the country-specific cluster variables and sampling weights. Adjusted estimates controlled for household wealth, rurality, and size; household head’s age and sex; maternal education, age, and age at first co-habitation; child age and sex; country and survey year. Abbreviations used: MD, mean difference; RR, relative risk.
