# Supplementary material for: Associations between women’s empowerment and child development, growth, and nurturing care practices in sub-Saharan Africa: A cross-sectional analysis of demographic and health survey data
Source: PLoS Med. 2021 Sep 16;18(9):e1003781. doi: 10.1371/journal.pmed.1003781 (PMC8483356; doi:10.1371/journal.pmed.1003781)
Supplement: S3 Table — Table A. Associations between women’s total empowerment and empowerment dimensions (continuous and quintile categories) and child outcomes, using the alternative definition of decision-making indicators. Table B. Associations between women’s total empowerment and empowerment dimensions (continuous and quintile categories) and care outcomes, using the alternative definition of decision-making indicators. (DOCX) [file pmed.1003781.s010.docx]

**S3 Table. Results using the alternative definition of decision-making indicators**

**Table A** Associations between women’s total empowerment and empowerment dimensions (continuous and quintile categories) and child outcomes, using the alternative definition of decision-making indicators^a^

|  | **Cognitive development off track**  **(N=20,019)**  **RR (95% CI)** | **Socio-emotional development off track**  **(N=19,688)**  **RR (95% CI)** | **Literacy-numeracy development off track**  **(N=19,335)**  **RR (95% CI)** | **Physical development off track**  **(N=20,082)**  **RR (95% CI)** | **Overall development off track**  **(N=19,255)**  **RR (95% CI)** |
| --- | --- | --- | --- | --- | --- |
| *Continuous* | | | | | |
| Total empowerment | 0.97 (0.96, 0.99) | 1.00 (0.98, 1.01) | 1.00 (1.00, 1.00) | 0.96 (0.93, 0.99) | 0.97 (0.94, 0.99) |
| Dimensions |  |  |  |  |  |
| Resources | 0.97 (0.94, 1.00) | 1.02 (0.99, 1.04) | 0.99 (0.99, 1.00) | 0.93 (0.89, 0.98) | 0.97 (0.93, 1.02) |
| Decision-making | 0.88 (0.81, 0.96) | 1.03 (0.96, 1.10) | 1.00 (0.98, 1.01) | 0.94 (0.81, 1.09) | 0.88 (0.78, 0.99) |
| Attitudes towards wife beating | 0.99 (0.97, 1.01) | 0.98 (0.96, 0.99) | 1.00 (1.00, 1.00) | 0.98 (0.95, 1.02) | 0.98 (0.95, 1.01) |
|  |  |  |  |  |  |
| *Quintile categories* | | | | | |
| Total empowerment |  |  |  |  |  |
| Empowerment Q1 (lowest) | Ref. | Ref. | Ref. | Ref. | Ref. |
| Empowerment Q2 | 0.95 (0.86, 1.04) | 1.03 (0.95, 1.12) | 0.99 (0.97, 1.01) | 0.89 (0.76, 1.04) | 0.84 (0.73, 0.96) |
| Empowerment Q3 | 0.96 (0.87, 1.05) | 0.96 (0.89, 1.04) | 0.99 (0.98, 1.01) | 0.87 (0.73, 1.04) | 0.84 (0.73, 0.97) |
| Empowerment Q4 | 0.92 (0.83, 1.02) | 0.97 (0.89, 1.05) | 0.98 (0.97, 1.00) | 0.78 (0.65, 0.94) | 0.84 (0.74, 0.96) |
| Empowerment Q5 (highest) | 0.88 (0.79, 0.98) | 1.01 (0.93, 1.10) | 0.99 (0.97, 1.00) | 0.81 (0.67, 0.97) | 0.82 (0.70, 0.96) |
| Dimensions |  |  |  |  |  |
| Resources Q1 (lowest) | Ref. | Ref. | Ref. | Ref. | Ref. |
| Resources Q2 | 0.89 (0.80, 0.99) | 0.93 (0.85, 1.01) | 1.00 (0.98, 1.02) | 0.95 (0.80, 1.13) | 0.85 (0.74, 0.98) |
| Resources Q3 | 0.96 (0.86, 1.07) | 0.97 (0.88, 1.07) | 0.99 (0.97, 1.01) | 1.00 (0.81, 1.22) | 0.87 (0.75, 1.00) |
| Resources Q4 | 1.00 (0.87, 1.13) | 0.92 (0.83, 1.02) | 1.01 (0.98, 1.03) | 0.87 (0.67, 1.13) | 0.91 (0.75, 1.11) |
| Resources Q5 (highest) | 0.92 (0.80, 1.04) | 0.98 (0.88, 1.08) | 0.98 (0.96, 1.01) | 0.92 (0.73, 1.15) | 0.87 (0.73, 1.03) |
| Decision-making Q1 (lowest) | Ref. | Ref. | Ref. | Ref. | Ref. |
| Decision-making Q2 | 0.81 (0.73, 0.91) | 0.98 (0.89, 1.07) | 1.00 (0.98, 1.01) | 0.73 (0.61, 0.89) | 0.76 (0.65, 0.88) |
| Decision-making Q3 | 0.77 (0.68, 0.88) | 0.97 (0.87, 1.07) | 0.98 (0.96, 1.00) | 0.69 (0.54, 0.88) | 0.69 (0.57, 0.84) |
| Decision-making Q4 | 0.79 (0.70, 0.90) | 0.98 (0.89, 1.08) | 0.99 (0.97, 1.02) | 0.78 (0.63, 0.97) | 0.82 (0.68, 0.98) |
| Decision-making Q5 (highest) | 0.79 (0.69, 0.91) | 1.03 (0.93, 1.14) | 0.99 (0.97, 1.01) | 0.70 (0.55, 0.90) | 0.78 (0.65, 0.94) |
| Attitudes towards wife beating Q1 (lowest) | Ref. | Ref. | Ref. | Ref. | Ref. |
| Attitudes towards wife beating Q2 | 1.00 (0.90, 1.10) | 1.05 (0.97, 1.14) | 1.01 (1.00, 1.03) | 1.29 (1.07, 1.56) | 1.08 (0.94, 1.25) |
| Attitudes towards wife beating Q3 | 0.82 (0.74, 0.92) | 1.01 (0.93, 1.09) | 0.99 (0.98, 1.01) | 1.05 (0.87, 1.28) | 0.86 (0.74, 0.99) |
| Attitudes towards wife beating Q4 | 0.96 (0.87, 1.06) | 0.98 (0.90, 1.07) | 1.00 (0.99, 1.02) | 0.90 (0.73, 1.11) | 0.96 (0.83, 1.11) |
| Attitudes towards wife beating Q5 (highest) | 0.96 (0.85, 1.08) | 0.90 (0.82, 0.99) | 1.01 (0.99, 1.03) | 1.32 (1.04, 1.66) | 1.01 (0.85, 1.20) |

|  | **Height-for-age Z-score**  **(N=20,390)**  **MD (95% CI)** | **Stunting (Height-for-age Z-score <-2)**  **(N=20,390)**  **RR (95% CI)** |
| --- | --- | --- |
| *Continuous* | | |
| Total empowerment | 0.01 (0.00, 0.02) | 0.99 (0.97, 1.00) |
| Dimensions |  |  |
| Resources | 0.03 (0.01, 0.05) | 0.97 (0.95, 0.99) |
| Decision-making | -0.03 (-0.08, 0.03) | 0.99 (0.95, 1.04) |
| Attitudes towards wife beating | 0.00 (-0.01, 0.02) | 1.00 (0.98, 1.01) |
|  |  |  |
| *Quintile categories* | | |
| Total empowerment |  |  |
| Empowerment Q1 (lowest) | Ref. | Ref. |
| Empowerment Q2 | -0.03 (-0.10, 0.04) | 1.01 (0.94, 1.07) |
| Empowerment Q3 | -0.03 (-0.10, 0.03) | 1.02 (0.96, 1.08) |
| Empowerment Q4 | 0.04 (-0.03, 0.11) | 0.99 (0.92, 1.06) |
| Empowerment Q5 (highest) | 0.11 (0.03, 0.18) | 0.90 (0.84, 0.97) |
| Dimensions |  |  |
| Resources Q1 (lowest) | Ref. | Ref. |
| Resources Q2 | -0.06 (-0.12, 0.01) | 1.04 (0.98, 1.10) |
| Resources Q3 | 0.02 (-0.06, 0.10) | 0.97 (0.90, 1.04) |
| Resources Q4 | 0.08 (-0.01, 0.17) | 0.94 (0.86, 1.02) |
| Resources Q5 (highest) | 0.11 (0.03, 0.20) | 0.91 (0.84, 0.98) |
| Decision-making Q1 (lowest) | Ref. | Ref. |
| Decision-making Q2 | -0.02 (-0.09, 0.05) | 1.05 (0.98, 1.12) |
| Decision-making Q3 | -0.02 (-0.11, 0.07) | 1.05 (0.97, 1.14) |
| Decision-making Q4 | -0.04 (-0.12, 0.04) | 1.01 (0.93, 1.09) |
| Decision-making Q5 (highest) | -0.04 (-0.12, 0.05) | 1.03 (0.95, 1.12) |
| Attitudes towards wife beating Q1 (lowest) | Ref. | Ref. |
| Attitudes towards wife beating Q2 | 0.02 (-0.05, 0.10) | 0.94 (0.88, 1.01) |
| Attitudes towards wife beating Q3 | 0.01 (-0.06, 0.09) | 0.99 (0.93, 1.06) |
| Attitudes towards wife beating Q4 | 0.04 (-0.03, 0.12) | 0.96 (0.90, 1.02) |
| Attitudes towards wife beating Q5 (highest) | -0.05 (-0.14, 0.04) | 1.03 (0.95, 1.11) |

^a^ In the primary decision-making definition, indicators on decision-making were coded as 1 if the woman alone or together with her husband/partner made the decision. In the alternative decision-making definition, indicators were coded as 1 if the woman alone made the decision. All estimates accounted for clustering and representativeness using the country-specific cluster variables and sampling weights, and controlled for household wealth, rurality, and size; household head’s age and sex; maternal education, age, and age at first co-habitation; child age and sex; country and survey year. Abbreviations used: Q, quintile category; RR, relative risk; MD, mean difference; Ref. reference.

**Table B** Associations between women’s total empowerment and empowerment dimensions (continuous and quintile categories) and care outcomes, using the alternative definition of decision-making indicators^a^

|  | **Number of learning resources (0-4)**  **(N=21,276)**  **MD (95% CI)** | **Number of maternal stimulation activities (0-6)**  **(N=20,745)**  **MD (95% CI)** | **≥4 maternal stimulation activities**  **(N=20,745)**  **RR (95% CI)** | **Number of paternal stimulation activities (0-6)**  **(N=20,745)**  **MD (95% CI)** | **≥4 paternal stimulation activities**  **(N=20,745)**  **RR (95% CI)** |
| --- | --- | --- | --- | --- | --- |
| *Continuous* | | | | | |
| Total empowerment | 0.01 (0.00, 0.02) | 0.01 (-0.01, 0.02) | 1.00 (0.97, 1.02) | 0.02 (0.02, 0.03) | 1.06 (1.02, 1.11) |
| Dimensions |  |  |  |  |  |
| Resources | 0.02 (0.01, 0.03) | 0.03 (0.00, 0.05) | 1.03 (0.99, 1.06) | 0.05 (0.03, 0.06) | 1.11 (1.04, 1.19) |
| Decision-making | 0.06 (0.01, 0.10) | 0.06 (-0.04, 0.15) | 1.13 (0.99, 1.30) | -0.14 (-0.18, -0.09) | 0.77 (0.56, 1.06) |
| Attitudes towards wife beating | 0.00 (-0.01, 0.01) | -0.01 (-0.04, 0.01) | 0.95 (0.92, 0.99) | 0.02 (0.01, 0.03) | 1.06 (0.99, 1.13) |
|  |  |  |  |  |  |
| *Quintile categories* | | | | | |
| Total empowerment |  |  |  |  |  |
| Empowerment Q1 (lowest) | Ref. | Ref. | Ref. | Ref. | Ref. |
| Empowerment Q2 | 0.07 (0.01, 0.12) | -0.01 (-0.10, 0.07) | 0.92 (0.80, 1.05) | 0.05 (-0.01, 0.10) | 1.03 (0.76, 1.41) |
| Empowerment Q3 | 0.01 (-0.04, 0.07) | 0.00 (-0.08, 0.08) | 0.89 (0.78, 1.02) | 0.10 (0.04, 0.15) | 1.18 (0.87, 1.59) |
| Empowerment Q4 | 0.09 (0.04, 0.14) | -0.02 (-0.12, 0.07) | 0.93 (0.81, 1.06) | 0.09 (0.03, 0.14) | 1.04 (0.79, 1.37) |
| Empowerment Q5 (highest) | 0.05 (-0.01, 0.12) | 0.05 (-0.05, 0.14) | 0.96 (0.83, 1.10) | 0.13 (0.07, 0.19) | 1.48 (1.11, 1.97) |
| Dimensions |  |  |  |  |  |
| Resources Q1 (lowest) | Ref. | Ref. | Ref. | Ref. | Ref. |
| Resources Q2 | 0.02 (-0.03, 0.08) | -0.09 (-0.18, 0.01) | 0.92 (0.79, 1.07) | 0.07 (0.01, 0.13) | 1.26 (0.90, 1.77) |
| Resources Q3 | 0.06 (-0.01, 0.12) | -0.18 (-0.30, -0.07) | 0.74 (0.62, 0.89) | 0.07 (0.01, 0.13) | 1.21 (0.86, 1.69) |
| Resources Q4 | -0.01 (-0.08, 0.06) | -0.11 (-0.23, 0.01) | 0.99 (0.84, 1.17) | 0.08 (0.01, 0.16) | 1.39 (0.96, 2.01) |
| Resources Q5 (highest) | 0.09 (0.02, 0.16) | -0.02 (-0.14, 0.10) | 0.99 (0.84, 1.17) | 0.16 (0.09, 0.24) | 1.73 (1.19, 2.50) |
| Decision-making Q1 (lowest) | Ref. | Ref. | Ref. | Ref. | Ref. |
| Decision-making Q2 | 0.07 (0.01, 0.13) | 0.00 (-0.09, 0.10) | 1.03 (0.88, 1.21) | 0.04 (-0.02, 0.11) | 0.95 (0.70, 1.29) |
| Decision-making Q3 | 0.04 (-0.04, 0.12) | 0.20 (0.08, 0.32) | 1.15 (0.95, 1.38) | 0.01 (-0.06, 0.08) | 0.91 (0.62, 1.32) |
| Decision-making Q4 | 0.07 (0.00, 0.15) | 0.24 (0.10, 0.38) | 1.26 (1.05, 1.53) | 0.03 (-0.04, 0.11) | 0.98 (0.68, 1.41) |
| Decision-making Q5 (highest) | 0.07 (0.00, 0.15) | 0.11 (-0.03, 0.25) | 1.22 (0.99, 1.51) | -0.15 (-0.22, -0.08) | 0.55 (0.35, 0.87) |
| Attitudes towards wife beating Q1 (lowest) | Ref. | Ref. | Ref. | Ref. | Ref. |
| Attitudes towards wife beating Q2 | -0.03 (-0.09, 0.03) | -0.22 (-0.33, -0.11) | 0.68 (0.58, 0.79) | 0.03 (-0.03, 0.09) | 1.02 (0.74, 1.41) |
| Attitudes towards wife beating Q3 | 0.03 (-0.03, 0.09) | -0.11 (-0.22, 0.00) | 0.84 (0.72, 0.98) | 0.07 (0.02, 0.12) | 1.27 (0.95, 1.70) |
| Attitudes towards wife beating Q4 | 0.02 (-0.04, 0.08) | -0.03 (-0.13, 0.08) | 0.86 (0.74, 1.00) | 0.09 (0.03, 0.14) | 1.16 (0.84, 1.59) |
| Attitudes towards wife beating Q5 (highest) | -0.04 (-0.11, 0.03) | -0.04 (-0.16, 0.07) | 0.81 (0.69, 0.95) | 0.09 (0.02, 0.15) | 1.01 (0.72, 1.43) |

|  | **Dietary diversity score (DDS, 0-7)**  **(N=11,279)**  **MD (95% CI)** | **Minimum dietary diversity (DDS≥4)**  **(N=11,279)**  **RR (95% CI)** |
| --- | --- | --- |
| *Continuous* | | |
| Total empowerment | 0.03 (0.02, 0.05) | 1.02 (1.00, 1.05) |
| Dimensions |  |  |
| Resources | 0.07 (0.04, 0.10) | 1.03 (0.99, 1.08) |
| Decision-making | 0.09 (-0.01, 0.19) | 1.06 (0.91, 1.24) |
| Attitudes towards wife beating | 0.00 (-0.02, 0.02) | 1.01 (0.98, 1.05) |
|  |  |  |
| *Quintile categories* | | |
| Total empowerment |  |  |
| Empowerment Q1 (lowest) | Ref. | Ref. |
| Empowerment Q2 | 0.04 (-0.08, 0.15) | 1.14 (0.96, 1.35) |
| Empowerment Q3 | 0.03 (-0.08, 0.14) | 1.07 (0.90, 1.27) |
| Empowerment Q4 | 0.07 (-0.04, 0.17) | 0.95 (0.80, 1.12) |
| Empowerment Q5 (highest) | 0.19 (0.06, 0.31) | 1.17 (0.99, 1.39) |
| Dimensions |  |  |
| Resources Q1 (lowest) | Ref. | Ref. |
| Resources Q2 | -0.01 (-0.13, 0.12) | 0.91 (0.75, 1.10) |
| Resources Q3 | 0.07 (-0.07, 0.20) | 1.07 (0.85, 1.33) |
| Resources Q4 | 0.02 (-0.13, 0.16) | 1.06 (0.83, 1.35) |
| Resources Q5 (highest) | 0.14 (-0.02, 0.29) | 0.99 (0.78, 1.25) |
| Decision-making Q1 (lowest) | Ref. | Ref. |
| Decision-making Q2 | -0.05 (-0.16, 0.06) | 0.81 (0.67, 0.99) |
| Decision-making Q3 | 0.09 (-0.05, 0.24) | 0.82 (0.65, 1.05) |
| Decision-making Q4 | 0.20 (0.04, 0.35) | 1.09 (0.88, 1.34) |
| Decision-making Q5 (highest) | 0.11 (-0.04, 0.27) | 0.94 (0.74, 1.19) |
| Attitudes towards wife beating Q1 (lowest) | Ref. | Ref. |
| Attitudes towards wife beating Q2 | -0.14 (-0.25, -0.03) | 0.93 (0.78, 1.11) |
| Attitudes towards wife beating Q3 | -0.07 (-0.18, 0.05) | 1.00 (0.84, 1.18) |
| Attitudes towards wife beating Q4 | -0.03 (-0.14, 0.07) | 0.91 (0.76, 1.08) |
| Attitudes towards wife beating Q5 (highest) | 0.04 (-0.09, 0.17) | 1.27 (1.05, 1.53) |

^a^ In the primary decision-making definition, indicators on decision-making were coded as 1 if the woman alone or together with her husband/partner made the decision. In the alternative decision-making definition, indicators were coded as 1 if the woman alone made the decision. All estimates accounted for clustering and representativeness using the country-specific cluster variables and sampling weights, and controlled for household wealth, rurality, and size; household head’s age and sex; maternal education, age, and age at first co-habitation; child age and sex; country and survey year. Abbreviations used: Q, quintile category; RR, relative risk; MD, mean difference; Ref. reference.
