# Supplementary material for: Associations between women’s empowerment and child development, growth, and nurturing care practices in sub-Saharan Africa: A cross-sectional analysis of demographic and health survey data
Source: PLoS Med. 2021 Sep 16;18(9):e1003781. doi: 10.1371/journal.pmed.1003781 (PMC8483356; doi:10.1371/journal.pmed.1003781)
Supplement: S4 Table — (DOCX) [file pmed.1003781.s011.docx]

**S10 Table** Effect heterogeneity of the association between women’s total empowerment and child and care outcomes by household wealth and maternal education, comparing women the highest quintile category to women in the lowest quintile category^a^

|  | **Cognitive development off track**  **(N=20,019)**  **RR (95% CI)** | **Socio-emotional development off track**  **(N=19,688)**  **RR (95% CI)** | **Learning-numeracy off track**  **(N=19,335)**  **RR (95% CI)** | **Physical development off track**  **(N=20,082)**  **RR (95% CI)** | **Overall development off track**  **(N=19,255)**  **RR (95% CI)** |
| --- | --- | --- | --- | --- | --- |
| Household wealth |  |  |  |  |  |
| Low wealth | 0.85 (0.74, 0.98) | 0.85 (0.74, 0.98) | 0.99 (0.97, 1.01) | 0.93 (0.70, 1.23) | 0.74 (0.60, 0.91) |
| High wealth | 0.90 (0.76, 1.06) | 0.97 (0.87, 1.09) | 0.93 (0.91, 0.96) | 0.74 (0.57, 0.98) | 0.94 (0.74, 1.19) |
| p-value for interaction | 0.607 | 0.148 | 0.001 | 0.262 | 0.127 |
| Maternal education |  |  |  |  |  |
| No education | 0.83 (0.73, 0.95) | 0.97 (0.86, 1.10) | 1.00 (0.98, 1.02) | 0.71 (0.55, 0.92) | 0.75 (0.62, 0.91) |
| Any education | 0.95 (0.80, 1.13) | 0.87 (0.78, 0.97) | 0.92 (0.89, 0.95) | 1.00 (0.74, 1.34) | 0.99 (0.76, 1.29) |
| p-value for interaction | 0.211 | 0.162 | <0.001 | 0.082 | 0.078 |
|  | **Height-for-age Z-score**  **(N=20,390)**  **MD (95% CI)** | **Stunting (Height-for-age Z-score <-2)**  **(N=20,390)**  **RR (95% CI)** |  |  |  |
| Household wealth |  |  |  |  |  |
| Low wealth | 0.16 (0.05, 0.27) | 0.93 (0.84, 1.02) |  |  |  |
| High wealth | 0.12 (0.03, 0.21) | 0.87 (0.79, 0.96) |  |  |  |
| p-value for interaction | 0.582 | 0.379 |  |  |  |
| Maternal education |  |  |  |  |  |
| No education | 0.10 (-0.00, 0.20) | 0.92 (0.85, 1.00) |  |  |  |
| Any education | 0.18 (0.09, 0.28) | 0.86 (0.77, 0.96) |  |  |  |
| p-value for interaction | 0.236 | 0.318 |  |  |  |
|  | **Number of learning resources (0-4)**  **(N=21,276)**  **MD (95% CI)** | **Number of maternal stimulation activities (0-6)**  **(N=20,745)**  **MD (95% CI)** | **≥4 maternal stimulation activities**  **(N=20,745)**  **RR (95% CI)** | **Number of paternal stimulation activities (0-6)**  **(N=20,745)**  **MD (95% CI)** | **≥4 paternal stimulation activities**  **(N=20,745)**  **RR (95% CI)** |
| Household wealth |  |  |  |  |  |
| Low wealth | 0.04 (-0.04, 0.12) | 0.11 (-0.04, 0.25) | 0.95 (0.74, 1.22) | 0.13 (0.05, 0.20) | 1.37 (0.90, 2.10) |
| High wealth | 0.15 (0.07, 0.24) | 0.29 (0.17, 0.42) | 1.23 (1.04, 1.46) | 0.35 (0.27, 0.43) | 2.47 (1.69, 3.61) |
| p-value for interaction | 0.047 | 0.055 | 0.089 | <0.001 | 0.039 |
| Maternal education |  |  |  |  |  |
| No education | 0.05 (-0.04, 0.13) | 0.04 (-0.10, 0.18) | 0.90 (0.71, 1.14) | 0.15 (0.09, 0.22) | 1.63 (1.01, 2.64) |
| Any education | 0.17 (0.10, 0.25) | 0.43 (0.30, 0.56) | 1.44 (1.22, 1.70) | 0.37 (0.27, 0.46) | 2.15 (1.53, 3.01) |
| p-value for interaction | 0.019 | <0.001 | 0.001 | <0.001 | 0.358 |
|  | **Dietary diversity score (DDS, 0-7)**  **(N=11,279)**  **MD (95% CI)** | **Minimum dietary diversity (DDS≥4)**  **(N=11,279)**  **RR (95% CI)** |  |  |  |
| Household wealth |  |  |  |  |  |
| Low wealth | 0.12 (-0.03, 0.26) | 1.13 (0.85, 1.50) |  |  |  |
| High wealth | 0.25 (0.09, 0.42) | 1.12 (0.92, 1.37) |  |  |  |
| p-value for interaction | 0.222 | 0.980 |  |  |  |
| Maternal education |  |  |  |  |  |
| No education | 0.18 (0.04, 0.32) | 1.03 (0.81, 1.31) |  |  |  |
| Any education | 0.21 (0.03, 0.40) | 1.21 (0.96, 1.51) |  |  |  |
| p-value for interaction | 0.784 | 0.345 |  |  |  |

^a^ All estimates accounted for clustering and representativeness using the country-specific cluster variables and sampling weights, and controlled for household wealth, rurality, and size; household head’s age and sex; maternal education, age, and age at first co-habitation; child age and sex; country and survey year. P-values for interaction presented throughout. Household wealth was entered as a binary variable, 1 = high wealth (household is in one of the highest three wealth quintiles) and 0 = low wealth (household is in one of the bottom two wealth quintiles). Maternal education was entered as a binary variable, 1 = any education and 0 = no education. Abbreviations used: RR, relative risk; MD, mean difference.
