# Supplementary material for: Associations between women’s empowerment and child development, growth, and nurturing care practices in sub-Saharan Africa: A cross-sectional analysis of demographic and health survey data
Source: PLoS Med. 2021 Sep 16;18(9):e1003781. doi: 10.1371/journal.pmed.1003781 (PMC8483356; doi:10.1371/journal.pmed.1003781)
Supplement: Attachment — Submitted filename: bliznashka.pdf [file pmed.1003781.s012.pdf]

This paper reports an investigation of empowerment and child development using a secondary data analysis.

I was asked for a statistical report and I interpret that to include all aspects of the design and conduct of the study.

Points of detail

Page 10 Naming factors is always a tricky issue but given the content of the items which go to make up the authors' gender dimension why was it not named 'intimate partner violence'? I am not an expert in this area but is there not more to gender roles than physical assault? Pages 22 and 23 seem to agree. Obviously if that is all DSS asks about then nothing can be done by the authors to broaden the scope of the factor analysis.

Pages 10 and 11 It is not clear to me why, if the authors had a theoretical model, they did not proceed straight to CFA to test it.

Page 13 There are only four quintiles. if 'fifth' lacks academic credentials then 'quintile category' might be better.

Page 13 According to Appendix S2 Table 2 there are only four binary indicators here for Gender so only $16 = 2^4$ possible values. I do not see how it makes sense to categorise the factor. Even if it does then categorising a variable wastes information (Altman and Royston, 2006; Royston et al., 2006) and leads to models which are often implausible as they predict the effect remaining flat within categories and then jumping to a new value at the category boundary. Decision making only has 32 possible values which is better but not ideal.

Page 13 Typo, link not like, I suppose.

Page 14 This tells us about missing data on confounders but what about missing data on the empowerment items?

Page 15 There does not seem to have been a formal test of the relationship between empowerment and any of the outcomes presented in Table 3. Just comparing two extreme groups is sub–optimal. This occurs elsewhere.

Page 15 Even if there is a relationship it only seems to be monotonic for the anthropometric outcomes not the other two which is worth comment. Similarly in Figure 2 for the sub–domains of empowerment many of these are not monotonic. It may be that it was expected that high and

low decision making empowerment would be best and middle worst, or vice versa, but has this been explained anywhere?

Page 15 It is better to state that the authors found no evidence for association rather than asserting that there was no association.

Page 17 Here we suddenly lose effect sizes with their confidence intervals and just see p–values which is a shame.

Page 21 In the absence of an overall test of the effect of empowerment on child growth I do not think it is wise to claim an effect here.

Page 26 'In conclusion, we showed that women's empowerment was positively associated with child cognitive development in SSA.' That is not the conclusion which I draw from Table 3 where the confidence intervals mostly include the null or from the parallel table in S8 Appendix where there is even less evidence.

S3 Appendix There seem to have been a number of places where the solutions differ between countries. This is not completely unexpected as the numbers per country cannot be large although I cannot find them in the submission. What are the implications of these differences for the underlying science here? Does this not throw some doubt on the existence of the constructs or their generalisability across cultures?

S3 Appendix, Table The rather high correlation between Factor 1 and 2 seems undesirable.

Points of more substance

Choice of latent variable method

Given that most of the manifest variables are binary and those which are not are ordered categorical I wonder why the authors did not use item response methods. This might have enabled inclusion of the items which were not asked in Congo as IRT methods cope with situations where not everybody answers all items. The use of differential item functioning might have meant that the scale could have been adapted to fit well across all the countries.

Descriptives

We are rather short on distributional information about the overall scores on the domains. In a study in which I was involved (not in one of the countries here) we had a very simple list of empowerment items and found the sum

score of positive responses was bi–modal: they either were empowered or they were not.

Summary

This is an extremely detailed report of the study but I have indicated some gaps in the analysis which make me feel that, as it stands, the authors' conclusions and interpretations go beyond what they have found in the analysis.

Michael Dewey

References

D G Altman and P Royston. The cost of dichotomising continuous variables. *British Medical Journal*, 332:1080, 2006.

P Royston, D G Altman, and W Sauerbrei. Dichotomizing continuous predictors in multiple regression: a bad idea. *Statistics in Medicine*, 25:127–141, 2006.
