# Supplementary material for: Associations between women’s empowerment and child development, growth, and nurturing care practices in sub-Saharan Africa: A cross-sectional analysis of demographic and health survey data
Source: PLoS Med. 2021 Sep 16;18(9):e1003781. doi: 10.1371/journal.pmed.1003781 (PMC8483356; doi:10.1371/journal.pmed.1003781)
Supplement: Attachment — Submitted filename: bliznashka2.pdf [file pmed.1003781.s014.pdf]

This paper reports an investigation of empowerment and child development using a secondary data analysis.

I was asked for a statistical report and I interpret that to include all aspects of the design and conduct of the study. When I am asked to look at a re–submission which I had already reviewed on a previous submission I restrict myself on principle to commenting on how the authors have responded to my original comments. I do not review the article again from scratch unless it has been so changed as to amount effectively to a new submission.

I said:

> *Naming factors is always a tricky issue but given the content of the items which go to make up the authors' gender dimension why was it not named 'intimate partner violence'? I am not an expert in this area but is there not more to gender roles than physical assault? Pages 22 and 23 seem to agree. Obviously if that is all DSS asks about then nothing can be done by the authors to broaden the scope of the factor analysis.*

The authors attempt to justify this in the rebuttal but really their statement there seems to amount to saying that they would have liked to have a factor embracing all the concepts they mention but they have not been able to have one because of limitations in the data but they still intend to call it Gender. I think it would be better to report what was actually found not what was desired to be found.

I said:

> *There are only four quintiles. if 'fifth' lacks academic credentials then 'quintile category' might be better.*

The authors mis–understand my point. There is one median which divides the sample into halves, two tertiles which divide the sample into thirds, three quartiles which divide the sample into quarters, and four quintiles which divide the sample into fifths. There is the potential for ambiguity here as quintile categories could be used in modelling as could the actual quintiles either as a categorical predictor or as a continuous one.

I said:

> *This tells us about missing data on confounders but what about missing data on the empowerment items?*

The authors state "Missing data on any of the exposure indicators or outcomes was not imputed to reduce the risk of bias."

It is complete cases analysis which introduces potential bias not multiple imputation. The authors' comment here does not justify ignoring the missingness.

The authors' discussion starts

> In this study, we found evidence suggesting that women's empowerment was positively associated with child cognitive development, growth, early learning, and nutrition outcomes among children 36-59 months of age in SSA.

I still feel that even in its toned down form that discussion over–interprets Table 3 and 4. Unless there was a prior hypothesis that just those categories would differ from the lowest on some but not all outcome variables then, in the absence of an overall test of difference, this is very data–driven.

Michael Dewey
