# Supplementary material for: Associations between women’s empowerment and child development, growth, and nurturing care practices in sub-Saharan Africa: A cross-sectional analysis of demographic and health survey data
Source: PLoS Med. 2021 Sep 16;18(9):e1003781. doi: 10.1371/journal.pmed.1003781 (PMC8483356; doi:10.1371/journal.pmed.1003781)
Supplement: Attachment — Submitted filename: R2 Responses to Reviewers.docx [file pmed.1003781.s013.docx]

We thank the Reviewers and the Editors for their time and thoughtful suggestions for improving our paper. We have provided point-by-point responses to their concerns and comments below.

**EDITORS**

**Please revise your title according to PLOS Medicine's style - ideally this should include a designation of the study design in the title (normally we recommend this is in the subtitle, after a colon) - here the study is based on DHS surveys, which are cross-sectional, correct? So one option might be: "Association between women’s empowerment and child development, growth, and nurturing care practices in sub-Saharan Africa: analysis of cross-sectional survey data".**

Thank you for this suggestion. We have revised the title to: *Associations between women’s empowerment and child development, growth, and nurturing care practices in sub-Saharan Africa: a cross-sectional analysis of Demographic and Health Survey data*

**In the last sentence of the Abstract Methods and Findings section, please include a note about any key limitation(s) of the study's methodology; in this case this might include the possibility for reverse causality or unmeasured confounding.**

Thank you for highlighting this omission. We have included key limitations in the abstract (lines 31-32): *“Study limitations include the possibility of reverse causality and suboptimal assessments of the outcomes and exposure.”*

**Did your study have a prospective protocol or analysis plan? Please state this (either way) early in the Methods section.**

We have provided the requested information in the Methods section (lines 201-207): *“We did not register a prospective analysis plan. However, all analyses were planned, and the only data-driven changes that occurred were in the derivation of the women’s empowerment score, as a result of conducting exploratory factor analysis. These were expected and are described in detail below. Changes in response to reviewers included external validation of the empowerment score and the inclusion of all four ECDI domains as outcomes. This study is reported according to the Strengthening the Reporting of Observational Studies in Epidemiology (STROBE) guidelines (S1 Checklist).”*

**As an analysis of cross-sectional survey data (the DHS surveys), we'd recommend that the authors use the STROBE guideline to support reporting of the study.**

We have included a completed STROBE checklist the Appendix.

**REVIEWER 1**

**This paper reports an investigation of empowerment and child development using a secondary data analysis. I was asked for a statistical report and I interpret that to include all aspects of the design and conduct of the study.**

Thank you for your detailed review and thoughtful suggestions for improving our paper.

**Page 10 Naming factors is always a tricky issue but given the content of the items which go to make up the authors' gender dimension why was it not named `intimate partner violence'? I am not an expert in this area but is there not more to gender roles than physical assault? Pages 22 and 23 seem to agree. Obviously if that is all DSS asks about then nothing can be done by the authors to broaden the scope of the factor analysis.**

Thank you for this suggestion. We have retained the factor name “Gender”. It is intended to capture the broader underlying construct rather than the individual indicators making up the factor score. We define the “Gender” dimension on lines 169-172 as *“Finally, the “Gender” dimension encompasses women’s intrinsic agency, i.e., voice and ability to express beliefs, and the extent to which women’s gender attitudes reflect normative gender beliefs including attitudes towards violence and beliefs around sexual activity [24].”* Thus, the construct captures more than attitudes towards wife-beating, which are the only indicators available in the DHS. We felt that using a narrower name for the factor may draw attention away from the breadth of gender norms this empowerment dimension is intended to capture. The DHS items we used assess women’s justification of violence which are intended to uncover underlying traditional beliefs about gender roles. In addition, DHS collect data on experience of intimate partner violence separately. Reducing confusion with whether we are talking about attitudes towards wife-beating or experience with intimate partner violence was another reason to retain the factor name “Gender”.

**Pages 10 and 11 It is not clear to me why, if the authors had a theoretical model, they did not proceed straight to CFA to test it.**

Thank you for this question. We have provided further rationale in the Methods section (lines 221-224): *“Despite our conceptual framework, using EFA prior to CFA allowed us to more fully explore the latent structure, verify that the three-factor solution had an acceptable fit and that indicator loadings were generally in line with our hypotheses [41]. EFA results helped refine the CFA specification.”* In addition, the empowerment score we conceptualized has not been previously tested in this form, and we therefore deemed EFA an appropriate first step.

**Page 13 There are only four quintiles. if `fifth' lacks academic credentials, then `quintile category' might be better.**

The total empowerment score and each dimension score were divided into quintiles; thus, there are five quintiles in each case. In the tables, we have listed quintile 1 (Q1) as the reference quintile. In the figures, only four quintiles are visible since each estimate is in reference to the first quintile. We have specified this in the footnotes to the figures.

**Page 13 According to Appendix S2 Table 2 there are only four binary indicators here for Gender so only 16 = 2^4^ possible values. I do not see how it makes sense to categorise the factor. Even if it does then categorising a variable wastes information (Altman and Royston, 2006; Royston et al., 2006) and leads to models which are often implausible as they predict the effect remaining at within categories and then jumping to a new value at the category boundary. Decision making only has 32 possible values which is better but not ideal.**

Thank you for raising this concern and providing these helpful references. We agree that dichotomising indicators could be problematic and have acknowledged this as a limitation (lines 575-579): *“In addition, similar to prior studies [24,67], we dichotomised most of the indicators used in our measurement model, which may lead to loss of information [68,69] and implausible models. This latter concern is addressed by our use of a strong conceptual framework, resulting in a form-invariant measurement model with acceptable psychometric properties, despite our use of binary indicators.”*

**Page 13 Typo, link not like, I suppose.**

Thank you for catching this typo. We have revised (lines 301-303): *“For continuous and count outcomes, we fit a generalized linear model and calculated unadjusted and adjusted mean differences (MD) and 95% confidence intervals (CIs).”*

**Page 14 This tells us about missing data on confounders but what about missing data on the empowerment items?**

Thank you for this question. We have specified how missing data on exposures and outcomes was handled in the Methods (lines 316-317): *“Missing data on any of the exposure indicators or outcomes was not imputed to reduce the risk of bias.”*

**Page 15 There does not seem to have been a formal test of the relationship between empowerment and any of the outcomes presented in Table 3. Just comparing two extreme groups is suboptimal. This occurs elsewhere.**

Thank you for raising this concern. We assessed the relationship between women’s empowerment and each outcome using biserial correlations. We now present these correlations in the S6 Appendix Empowerment Score Diagnostics. We refer the reader to this appendix on lines 299-301: *“Biserial correlations between individual dimensions and total empowerment scores and quintiles and each outcome are presented in* ***Tables 1 and 2 in S6 Appendix****.”*

**Page 15 Even if there is a relationship it only seems to be monotonic for the anthropometric outcomes not the other two which is worth comment. Similarly in Figure 2 for the sub-domains of empowerment many of these are not monotonic. It may be that it was expected that high and low decision making empowerment would be best and middle worst, or vice versa, but has this been explained anywhere?**

Thank you for raising this concern. We have commented on this in the Discussion (lines 442-445): *“Surprisingly, associations between women’s empowerment and child development were not monotonic, i.e., the magnitude of the associations was similar across quintiles of women’s empowerment and its dimensions. In contrast, associations between women’s empowerment and parental stimulation generally increased in higher empowerment quintiles.”* We refrain from commenting on the relationship for anthropometric outcomes since we only find a statistically significant association for HAZ and stunting in the highest empowerment quintile. The remaining CIs are wide and cross the null, and we cannot say with sufficient certainty that the relationships are monotonic.

**Page 15 It is better to state that the authors found no evidence for association rather than asserting that there was no association.**

Thank you, we have revised accordingly throughout the Results and Discussion.

**Page 17 Here we suddenly lose effect sizes with their confidence intervals and just see p-values which is a shame.**

We have now provided the effect estimates and 95% CIs together with the p-values for interaction in the main text. Please see the revised Heterogeneity sections in the Results, lines 403-427.

**Page 21 In the absence of an overall test of the effect of empowerment on child growth I do not think it is wise to claim an effect here.**

Thank you for raising this concern. We agree that our statement was strongly worded and have revised accordingly (lines 429-433): *“In this study, we found evidence suggesting that women’s empowerment was positively associated with child cognitive development, growth, early learning, and nutrition outcomes among children 36-59 months of age in SSA. However, with respect to child growth and nutrition, evidence was limited with associations significant only among women in the highest empowerment quintile, relative to the lowest.”*

**Page 26 `In conclusion, we showed that women's empowerment was positively associated with child cognitive development in SSA.' That is not the conclusion which I draw from Table 3 where the confidence intervals mostly include the null or from the parallel table in S8 Appendix where there is even less evidence.**

Thank you for raising this concern. In the revised manuscript, we included two additional development domains, which changed our sample size for the cognitive and socio-emotional development a little bit. The CIs for quintiles 3, 4 and 5, relative to the lowest, are below the null. We believe our conclusion is supported by these results. However, we have phrased it more cautiously (lines 667-669): *“In conclusion, we found evidence suggesting that women’s empowerment was positively associated with child cognitive development in SSA, but not child socio-emotional, literacy-numeracy, or physical development.”*

**S3 Appendix There seem to have been a number of places where the solutions differed between countries. This is not completely unexpected as the numbers per country cannot be large although I cannot find them in the submission. What are the implications of these differences for the underlying science here? Does this not throw some doubt on the existence of the constructs or their generalisability across cultures?**

Thank you for raising these concerns. We have now included the overall sample size for each country in the Methods section (lines 181-186). Our EFA and CFA solutions did indeed differ by country, which led to a reduced three-factor form-invariant model. We have now commented on the implications in the Discussion (lines 614-617): *“Indeed, our results showed that the “access to healthcare” dimension may be context-specific as this factor did not emerge in two of the countries in our sample. Although excluding this dimension may limit the scope of our empowerment score, it may increase its generalisability because it only reflects universal aspects of women’s empowerment.”*

**S3 Appendix, Table The rather high correlation between Factor 1 and 2 seems undesirable.**

We agree that the 0.407 correlation between factors 1 and 2 in the three-factor solution is relatively high compared with the correlations between factors 1 and 3 and factors 2 and 3. However, it is far below our *a priori* defined 0.8 threshold indicating poor discriminant validity and typically used in the literature (Brown, 2006b). We specify this threshold in S2 Appendix: *“Factor correlations were used to evaluate discriminant validity with correlations ≥0.8 indicating poor discriminant validity and suggesting a more parsimonious solution [79].”*

Reference:

Brown TA. The Common Factor Model and Exploratory Factor Analysis. First. Confirmatory Factor Analysis for Applied Research. First. New York: The Guilford Press; 2006. pp. 12–39.

**Choice of latent variable method**

**Given that most of the manifest variables are binary and those which are not are ordered categorical I wonder why the authors did not use item response methods. This might have enabled inclusion of the items which were not asked in Congo as IRT methods cope with situations where not everybody answers all items. The use of differential item functioning might have meant that the scale could have been adapted to fit well across all the countries.**

Thank you for this question. A unidimensional two-parameter normal ogive IRT model and a one-factor non-linear CFA with binary and categorical variables are formally equivalent. The only crucial difference is where the marginalization is performed (over the number of factors or over the number of items) which determines the use of different optimization approaches (i.e., maximum likelihood in the case of IRT and generalised lest squares in the case of CFA) (Takane & de Leeuw, 1987). Both methods are appropriate for assessing measurement invariance. However, CFA is recommended/preferred in multifactorial frameworks (Meade & Lautenschlager, 2004; Wirth & Edwards, 2007), when the number of items is small, and when many groups are compared (Meade & Lautenschlager, 2004). In practice, CFA is more frequently used than IRT when a multidimensional structure is of interest (ten Holt et al., 2010). For these reasons, we used CFA instead of IRT. We have now provided this rationale in the manuscript (lines 213-216): *“Although factor analysis and item response theory with binary and categorical variables are formally equivalent, factor analysis is preferred in multidimensional frameworks, with a small number of items, and when several groups are compared for measurement invariance [38–40].”*

References:

Meade AW, Lautenschlager GJ. A Comparison of Item Response Theory and Confirmatory Factor Analytic Methodologies for Establishing Measurement Equivalence/Invariance. Organ Res Methods 2004;7:361–88. <https://doi.org/10.1177/1094428104268027>

Takane Y, de Leeuw J. On the relationship between item response theory and factor analysis of discretized variables. Psychometrika 1987;52:393–408. <https://doi.org/10.1007/BF02294363>.

ten Holt JC, van Duijn MAJ, Boomsma A. Scale construction and evaluation in practice: A review of factor analysis versus item response theory applications. Psychol Test Assess Model 2010;52:272–297.

Wirth RJ, Edwards MC. Item factor analysis: Current approaches and future directions. Psychol Methods 2007;12:58–79. <https://doi.org/10.1037/1082-989X.12.1.58>

**Descriptives**

**We are rather short on distributional information about the overall scores on the domains. In a study in which I was involved (not in one of the countries here) we had a very simple list of empowerment items and found the sum score of positive responses was bi-modal: they either were empowered or they were not.**

Thank you for raising this point. We have included the distributions of the individual and overall scores in a new appendix S6 Appendix. We refer the readers to the histograms on lines 240-242: *“The distributions of the individual dimension factor scores and the total empowerment score are shown in* ***Figure 1 in S6 Appendix.”*** Overall, the scores appeared normally distributed. There was some suggestive evidence that the Gender dimension score might not be normally distributed. We tried a log transformation, but that did not make the gender score more normally distributed. We left the factor scores as estimated from the measurement model for ease of interpretation, i.e., in terms of changes in SD units.

**Summary**

**This is an extremely detailed report of the study but I have indicated some gaps in the analysis which make me feel that, as it stands, the authors' conclusions and interpretations go beyond what they have found in the analysis.**

Thank you for your thorough and thoughtful comments. We believe that in addressing them we have greatly improved the paper and in particular the clarity of the methods, results and discussion sections.

-----------------------------------------------------------

**REVIEWER 2**

**Overall, this study is excellent study and the manuscript well-written. The methods and interpretation were thoughtful and meticulously presented.**

Thank you for your detailed review and thoughtful suggestions for improving our paper.

**1.      Line 152. Use "resources" for brevity rather than "access" for two reasons. One, both access and control are important.  Two, then this language would better articulate with Kabeer and with the concept of resources for care in the UNICEF extended model for care (reference 19).**

Thank you for this suggestion! We have made the change throughout the text.

**2.      Line 261.  "with an identity like"?  Authors meant "link"? Why not just say "we fit a general linear model"?**

Thank you for catching this typo. We have revised (lines 301-303): *“For continuous and count outcomes, we fit a generalized linear model and calculated unadjusted and adjusted mean differences (MD) and 95% confidence intervals (CIs).”*

**3.      Lines 377-378. Prior research with MICS data has shown that the socio-emotional indicators in the ECDI are not reliable at the individual level. That knowledge should be brought into the discussion here.  See the ECDI measures section of: Frongillo EA, Kulkarni S, Basnet S, de Castro F.  Family care behaviors and early childhood development in low- and middle-income countries. Journal of Child and Family Studies 26:3036-3044, 2017.**

Thank you for raising this point and providing this helpful reference. We have reflected this point in the Discussion (lines 465-472): *“With respect to socio-emotional and physical development, the associations with women’s empowerment and its dimensions were limited. Given the positive associations between women’s empowerment and early learning, it is possible that ECDI may not adequately capture socio-emotional development (with items focused on behavioural challenges) and non-differential outcome misclassification would bias the associations with women’s empowerment toward the null. In addition, prior studies have shown that the physical and socio-emotional indicators of the ECDI are not reliable at the individual level [55].”*

-----------------------------------------------------------

**REVIEWER 3**

**1.      This is an important analysis that contributes to the growing literature on women's empowerment using survey data.**

Thank you for your detailed review and thoughtful suggestions for improving our paper.

**2.      The literature review on which the article is based seems outdated, however. The authors cite a "large body of literature" on women's empowerment and child health and nutrition (l.81), but the latest article is from 2016. A lot has happened since then.**

Thank you for raising this point. The latest article we had cited was Santoso et al. 2019, which was the latest systematic review we were aware of at the time of submission (November 2020). We have now included another recently published systematic review by Abreha et al. on the relationship between women’s empowerment and child health, as well as two empirical articles on women’s empowerment and child survival (Doku et al. 2020, Panday et al. 2020) and one article on women’s empowerment and women’s nutritional status (Jones et al. 2020).

Reference:

Abreha SK, Zereyesus YA. Women’s Empowerment and Infant and Child Health Status in Sub-Saharan Africa: A Systematic Review. Matern Child Health J 2020. <https://doi.org/10.1007/s10995-020-03025-y>.

Doku DT, Bhutta ZA, Neupane S. Associations of women’s empowerment with neonatal, infant and under-5 mortality in low- and /middle-income countries: meta-analysis of individual participant data from 59 countries. BMJ Glob Heal 2020;5:e001558. <https://doi.org/10.1136/bmjgh-2019-001558>.

Jones RE, Haardörfer R, Ramakrishnan U, Yount KM, Miedema SS, Roach TD, et al. Intrinsic and instrumental agency associated with nutritional status of East African women. Soc Sci Med 2020;247:112803. <https://doi.org/10.1016/j.socscimed.2020.112803>.

Panday P, Rackie D, Kula MC. The status of women and its influence on children’s well‐being: Do geography, religion and income matter? A comparative study. Dev Policy Rev 2020;38:766–82. <https://doi.org/10.1111/dpr.12472>.

**3.      Next, the authors claim that no study has looked at the association between empowerment and child development. It's true for the limited list of articles cited. But they missed "The impact of women's empowerment on their children's early development in 26 African countries." published in the Journal of Global Health,**  [**https://doi.org/10.7189/jogh.10.020406**](https://doi.org/10.7189/jogh.10.020406)**.**

Thank you for highlighting this recently published study and providing us with the reference. We were not aware of this study at the time of submission (November 2020). We have now referenced it in the Introduction (lines 88-91): *“Likewise, only one study has examined the association between women’s empowerment and child development in SSA [20]. This study showed that women’s empowerment was positively associated with child literacy-numeracy development, but not with child cognitive, socio-emotional or physical development [20].”* and in the Discussion (lines 446-460): *“To our knowledge, this is only the second study to provide evidence on the association between women’s empowerment and child development. Another recent study, which pooled DHS and MICS data from 26 African countries, showed that different aspects of women’s empowerment were positively associated with different domains of child development in SSA [20]. Similar to Ewerling et al., we found that women’s decision-making empowerment predicted better cognitive development. However, in contrast to this study, we found no evidence that women’s empowerment or its dimensions was associated with child literacy-numeracy development, and limited evidence that women’s gender empowerment was associated with socio-emotional development. Differences in the operationalization of women’s empowerment dimensions, the statistical methods employed, and the sample of countries (in addition to including more SSA countries, Ewerling et al. also included two North African countries) may help explain the differences in findings between the two studies. Of note is that Ewerling et al. impute at least some exposure information for nearly 40% of their sample. Although cautiously conducted, this imputation could have biased the association between women’s empowerment and child development.”*

**4.      In short, the introduction misses several more recent publications on women's empowerment and several other aspects of child health and survival using survey data, giving the reader the false impression that the topic is understudied.**

Thank you for raising this concern. We had previously included all systematic reviews on women’s empowerment and child outcomes in low- and middle-income countries up to 2019. We have now included another recently published systematic review on women’s empowerment and child health in sub-Saharan Africa, published after our submission. We have also included two studies examining the relationship between women’s empowerment and child survival (Doku et al. 2020, Panday et al. 2020). We have revised the Introduction to clarify that the topic of women’s empowerment and child survival, health, nutrition, and growth is indeed well studied. However, the topic of women’s empowerment and child development, responsive care, early learning opportunities, and safety and security (as defined in the Children Surviving and Thrive Framework) have remained understudied.

*“However, where a large body of literature from LMICs has examined the associations between caregiver empowerment and child survival, health, nutrition, and growth [11–19], associations with other proximal components of the Children Surviving and Thriving Framework remain understudied. For instance, to our knowledge, no evidence exists on whether women’s empowerment is associated with responsive care, early learning opportunities, and security and safety. Likewise, only one study has examined the association between women’s empowerment and child development in SSA [20]. This study showed that women’s empowerment was positively associated with child literacy-numeracy development, but not with child cognitive, socio-emotional or physical development [20].”* (lines 83-91)

**5.      Figure 1 shows pathways through which empowerment would affect child development (and growth? not included in the caption). The first thing that calls my attention is that many of the arrows are bidirectional. I find it hard to interpret, and mostly to implement in any type of analysis. I also do not recognize the most commonly described dimensions of empowerment in this model.**

Thank you for raising these concerns. We have simplified Figure 1 to reduce the number of boxes and arrows. We have also added a legend, explaining the bidirectional arrows. In addition, we have clarified in the text why some arrows are bi-directional (lines 155-160): *“Of note is that these pathways are interrelated and the temporal order in which they influence each other is unclear. In addition, the causal direction between women’s empowerment and women’s and children’s health and nutrition outcomes in our cross-sectional model is ambiguous. For example, women’s empowerment may help improve child health outcomes or improved child health may empower women. We express such interactive relationships via bi-directional arrows in Fig 1.”*

As to the naming of the dimensions, we provide a summary of definitions and measurements of women’s empowerment in S1 Appendix. Although the precise language defers across studies, we believe our labelling of the dimensions as “Resources”, “Decision-making”, and “Gender attitudes” is in fact in line with the literature and specifically with studies grounded in Kabeer’s framework. For instance, similarly, Miedema and colleagues define a “gender beliefs and attitudes” and a “household decision-making dimension. Asaolu and colleagues define an “attitudes towards violence” and a “work/labour force participation” dimension, the former including indicators in our “Gender attitudes” dimension and the latter including indicators in our “Resources” dimension. Ewerling and colleagues define an “attitudes towards violence” (indicators we include in our “Gender attitudes” dimension) and a “decision-making” dimension. Our naming is intended to reflect the underlying construct, i.e., gender attitudes, rather than the indicators that make up the factor score, i.e., attitudes towards wife-beating, which are subject to data availability given that we are working with secondary data.

References:

Asaolu IO, Alaofè H, Gunn JKL, Adu AK, Monroy AJ, Ehiri JE, et al. Measuring Women’s Empowerment in Sub-Saharan Africa: Exploratory and Confirmatory Factor Analyses of the Demographic and Health Surveys. Front Psychol 2018;9. <https://doi.org/10.3389/fpsyg.2018.00994>.

Ewerling F, Lynch JW, Victora CG, van Eerdewijk A, Tyszler M, Barros AJD. The SWPER index for women’s empowerment in Africa: development and validation of an index based on survey data. Lancet Glob Heal 2017;5:e916–23. <https://doi.org/10.1016/S2214-109X(17)30292-9>.

Miedema SS, Haardörfer R, Girard AW, Yount KM. Women’s empowerment in East Africa: Development of a cross-country comparable measure. World Dev 2018;110:453–64. <https://doi.org/10.1016/j.worlddev.2018.05.031>.

**6.      It is not clear from the methods section why only DHS surveys were used in the analysis. The decision is surprising given that MICS was the survey family that started applying the ECDI module, and using MICS surveys would largely increase the number of countries represented in the SSA region.**

Thank you for raising this concern. We have provided our rationale in the Methods (lines 198-200): *“Although the MICS also collect data on child development, growth, and early learning, they do not collect data on women’s decision-making in the household or on child diet for children 36-59 months of age. Therefore, we did not include MICS data.”* We have also recognized this as a limitation in the Discussion (lines 617-620): *“Nevertheless, we were limited to nine SSA countries as we excluded MICS data which do not collect information on household decision-making or child diet. Thus, we were unable to assess if our findings extend to other SSA countries.”* Although Ewerling and colleagues pool DHS and MICS data in their recent article on women’s empowerment and child development, kindly shared by the Reviewer, they reported imputing some data for 38% of women in their sample which was primarily the MICS survey data due to the lack of data on household decision-making. Imputing data for both the exposure and the outcome could lead to high risk of bias, and we therefore chose not to impute this data in our study. We have now specified this reasoning in the Methods (lines 316-317): *“Missing data on any of the exposure indicators or outcomes was not imputed to reduce the risk of bias.”*

**7.      Regarding the empowerment indicator used, despite the criticisms raised by the authors against other attempts, they provide details on what was developed in a series of appendices. The indicator was not published and subject to direct screening by peers. The review of the indicator would deserve a full evaluation. In my opinion the validation presented relies strongly in psychometric approaches, where some ideal statistics are sought. But we do not see any evidence that the resulting scores have any meaning as measures of empowerment in the form of some external validation with other existing indicators.**

Thank you for raising these concerns. We had previously reported all statistics for the psychometric methods we used in the appendices. We have now moved the fit statistics for the final form-invariant measurement model to the main text (lines 233-236): *“The final form-invariant measurement model showed acceptable model fit, based on a priori determined acceptability thresholds [43]: Comparative Fit Index of 0.973 (threshold ≥ 0.95), Root Mean Square Error of Approximation of 0.043 (threshold ≤ 0.08), and Root Mean Square Error of Approximation of 0.055 (threshold ≤ 0.08).”*

We have also provided details on the external validation we conducted in the Methods section (lines 243-253): *“External validity was assessed against the United Nations Gender Inequality Index (GII). The GII is a country-level index that measures gender inequity in reproductive health, political empowerment, and economic activity. A higher GII value indicates more gender disparity [44], and we therefore expected a negative correlation between our empowerment measures and the GII. We estimated country-level means for the individual dimension and total empowerment scores, adjusting for the complex survey design using country-specific sampling weights and clustering variables. The correlations with the GII were -0.633 (p=0.068) for total empowerment, -0.711 (p=0.032) for “Resources”, -0.642 (p=0.062) for “Decision-making”, and -0.343 (p=0.366) for “Gender” (Figure 2 in S6 Appendix). The strongest, statistically significant correlation with “Resources” was expected given that women’s labour force participation is the only common indicator between the GII and our empowerment score (part of the “Resources” dimension).”*

We have also highlighted the need for common approaches to assessing women’s empowerment in the Discussion (lines 621-622): *“Common measurement, validation, and analysis approaches of women’s empowerment in diverse contexts are needed.”*

**8.      The ECDI has been heavily criticized in the literature, and alternative ways to assessing child development have been proposed, besides the original proposal. Here, the authors decided to drop two of the original domains and keep the social-emotional and cognitive domains. The reasons presented, that one set of items is too difficult and the other is too easy do not convince me. Checking the proportion of children failing these dimensions confirm what the authors say, but in including these items, the indicator as a whole has more information to discriminate children that are not on track. In my opinion, dropping information from what is available only makes the indicator weaker. The decision basically relies on one publication that is very critical of the indicator. If the authors agree with that paper, one wonders if the indicators should have been used here at all.**

Thank you for raising this important point. We agree that in excluding the literacy-numeracy and physical domains we may be losing important information and possibly making the indicator weaker. Therefore, we have included the literacy-numeracy and physical domains in the revised manuscript. We have re-calculated the total development indicator to account for all four domains. We have revised the Methods section accordingly. We now present the associations between women’s empowerment and literacy-numeracy and physical development in the Results section. Interpretations and implications are summarized in the Discussion. For brevity, we refer the Reviewer to the revised manuscript for all these changes.

**9.      The empowerment scores were divided into quintiles. It would be interesting to have an idea of the score distribution. FA scores based on dichotomous variables can pile up in a few values and make the use of quintiles very hard to interpret.**

Thank you for raising this point. We have included the distributions of the individual and overall scores in a new appendix S6 Appendix. Empowerment Score Diagnostics. We refer the readers to the histograms on lines 240-242: *“The distributions of the individual dimension factor scores and the total empowerment score are shown in* ***Figure 1 in S6 Appendix.”*** Overall, the scores appeared normally distributed. There was some suggestive evidence that the Gender dimension score might not be normally distributed. We tried a log transformation, but that did not make the gender score more normally distributed. We left the factor scores as estimated from the measurement model for ease of interpretation, i.e., in terms of changes in SD units.

**10.     It would be good to cite a source justifying the use of a Poisson model for binary outcomes, even if this practice is quite common currently.**

We have now provided a reference on line 304.

**11.     The list of confounders include, in my opinion, several variable that actually constitute aspects of empowerment, like education and age at first marriage.**

Thank you for raising this concern. We have now clarified our rationale for treating women’s education and age at first co-habitation as covariates in the Methods section (lines 309-314): *“Although some measures of women’s empowerment include woman’s education and age at first co-habitation as indicators [24,51], we excluded them from the empowerment score and treated them as covariates for two main reasons. First, it is unclear if women’s education and age at first co-habitation represent resources for empowerment, achievement of the empowerment process, or both [52]. Second, women’s education is often considered an indicator of women’s status and is relatively fixed in adulthood [53].”*

In addition, we ran sensitivity analyses excluding women’s education and age at first co-habitation as covariates. Our results changed little after conducting this sensitivity analyses. For brevity, we only show associations between women’s empowerment and child development. However, our findings were robust across the remaining outcomes. Although for brevity we have chosen not to include these findings in the manuscript itself, we provide here a table for the Reviewer’s convenience. If the Reviewer and Editors feel that providing the results excluding women’s education and age at first co-habitation as covariates can greatly improve the manuscript, we ask that you let us know and we can include them in the appendix.

|  | **Full set of covariates** | | | | | **Reduced set of covaries, excluding women’s education and age at first co-habitation** | | | |
| --- | --- | --- | --- | --- | --- | --- | --- | --- | --- |
|  | **Cognitive development off track**  **(N=20,019)**  **RR (95% CI)** | **Socio-emotional development off track**  **(N=19,688)**  **RR (95% CI)** | **Learning-numeracy off track**  **(N=19,335)**  **RR (95% CI)** | **Physical development off track**  **(N=20,082)**  **RR (95% CI)** | | **Cognitive development off track**  **(N=20,019)**  **RR (95% CI)** | **Socio-emotional development off track**  **(N=19,688)**  **RR (95% CI)** | **Learning-numeracy off track**  **(N=19,335)**  **RR (95% CI)** | **Physical development off track**  **(N=20,082)**  **RR (95% CI)** |
| Empowerment Q2 | 0.91 (0.82, 1) | 1.00 (0.92, 1.09) | 1.00 (0.98, 1.01) | 1.06 (0.88, 1.27) | | 0.9 (0.82, 0.99) | 1 (0.92, 1.09) | 0.99 (0.98, 1.01) | 1.05 (0.87, 1.26) |
| Empowerment Q3 | 0.88 (0.8, 0.98) | 0.93 (0.86, 1.01) | 0.99 (0.98, 1.01) | 0.91 (0.75, 1.1) | | 0.87 (0.79, 0.97) | 0.93 (0.86, 1.01) | 0.99 (0.97, 1) | 0.89 (0.73, 1.08) |
| Empowerment Q4 | 0.89 (0.81, 0.98) | 0.97 (0.89, 1.05) | 0.99 (0.97, 1) | 0.82 (0.67, 0.99) | | 0.87 (0.79, 0.96) | 0.97 (0.89, 1.05) | 0.97 (0.96, 0.99) | 0.78 (0.65, 0.95) |
| Empowerment Q5 | 0.89 (0.8, 0.99) | 0.93 (0.85, 1.02) | 0.98 (0.96, 1) | 0.83 (0.68, 1.02) | | 0.86 (0.77, 0.96) | 0.93 (0.85, 1.02) | 0.96 (0.94, 0.98) | 0.78 (0.64, 0.96) |
|  |  |  |  |  |  |  |  |  |  |
| Access Q2 | 0.92 (0.83, 1.03) | 0.88 (0.8, 0.96) | 0.99 (0.97, 1.01) | 0.94 (0.79, 1.12) | | 0.93 (0.83, 1.03) | 0.88 (0.8, 0.96) | 1 (0.98, 1.01) | 0.93 (0.79, 1.11) |
| Access Q3 | 0.96 (0.87, 1.07) | 0.94 (0.86, 1.04) | 1 (0.98, 1.02) | 0.86 (0.72, 1.03) | | 0.97 (0.87, 1.08) | 0.94 (0.86, 1.04) | 1 (0.98, 1.02) | 0.86 (0.72, 1.03) |
| Access Q4 | 0.88 (0.78, 1) | 0.89 (0.81, 0.98) | 0.98 (0.96, 1) | 0.73 (0.59, 0.9) | | 0.88 (0.78, 0.99) | 0.89 (0.81, 0.98) | 0.98 (0.96, 1.01) | 0.71 (0.58, 0.88) |
| Access Q5 | 0.83 (0.72, 0.95) | 0.93 (0.84, 1.04) | 0.99 (0.96, 1.01) | 0.79 (0.62, 1.01) | | 0.81 (0.71, 0.94) | 0.94 (0.84, 1.04) | 0.98 (0.96, 1.01) | 0.76 (0.59, 0.96) |
| Decision-making Q2 | 0.89 (0.8, 0.99) | 0.94 (0.86, 1.02) | 0.99 (0.98, 1.01) | 0.8 (0.67, 0.96) | | 0.88 (0.79, 0.98) | 0.94 (0.86, 1.02) | 0.99 (0.97, 1.01) | 0.8 (0.66, 0.96) |
| Decision-making Q3 | 0.89 (0.8, 0.99) | 0.98 (0.89, 1.08) | 1 (0.98, 1.02) | 0.94 (0.77, 1.14) | | 0.88 (0.79, 0.98) | 0.98 (0.89, 1.08) | 1 (0.97, 1.02) | 0.93 (0.76, 1.13) |
| Decision-making Q4 | 0.81 (0.71, 0.93) | 0.97 (0.88, 1.08) | 0.99 (0.97, 1.01) | 0.68 (0.53, 0.85) | | 0.8 (0.7, 0.92) | 0.98 (0.88, 1.09) | 0.98 (0.96, 1.01) | 0.67 (0.53, 0.84) |
| Decision-making Q5 | 0.92 (0.79, 1.06) | 0.99 (0.88, 1.11) | 0.98 (0.96, 1) | 0.88 (0.67, 1.17) | | 0.9 (0.78, 1.04) | 0.99 (0.88, 1.11) | 0.97 (0.95, 0.99) | 0.87 (0.65, 1.15) |
| Gender Q2 | 0.97 (0.89, 1.07) | 1.04 (0.96, 1.13) | 1.01 (1, 1.03) | 1.09 (0.9, 1.31) | | 0.97 (0.88, 1.06) | 1.04 (0.96, 1.13) | 1.01 (0.99, 1.03) | 1.08 (0.89, 1.3) |
| Gender Q3 | 0.82 (0.74, 0.91) | 0.98 (0.9, 1.06) | 1 (0.99, 1.02) | 0.95 (0.79, 1.14) | | 0.82 (0.74, 0.91) | 0.98 (0.9, 1.06) | 1 (0.99, 1.02) | 0.94 (0.78, 1.13) |
| Gender Q4 | 0.97 (0.87, 1.07) | 1.02 (0.94, 1.12) | 1.01 (0.99, 1.03) | 0.99 (0.81, 1.21) | | 0.96 (0.87, 1.06) | 1.02 (0.94, 1.12) | 1 (0.98, 1.02) | 0.98 (0.8, 1.21) |
| Gender Q5 | 0.97 (0.85, 1.11) | 0.84 (0.76, 0.94) | 1.01 (0.99, 1.03) | 1.15 (0.9, 1.47) | | 0.97 (0.84, 1.1) | 0.84 (0.76, 0.94) | 1 (0.98, 1.02) | 1.14 (0.89, 1.46) |

All estimates accounted for clustering and representativeness using the country-specific cluster variables and sampling weights. The full set of covariates included household wealth, rurality, and size; household head’s age and sex; maternal education, age, and age at first co-habitation; child age and sex; country and survey year. The reduced set of covariates included household wealth, rurality, and size; household head’s age and sex; maternal age; child age and sex; country and survey year. Abbreviations used: RR, relative risk; MD, mean difference.

**12.     I find the presentation of results hard to follow. Especially figures 2 and 3, with very large Cis and no clear trend, do not help understand whatever the data may be suggesting. It is not specified in the figures that the development indicators are actually children not on track. A less careful examination of the figures suggest that empowerment is detrimental to development.**

We have revised the Results to improve the flow. For brevity we refer the Reviewer to the manuscript. We have also revised all figures with development outcomes to now specify the indicators are for children developmentally off track. Regarding the CIs, we would like to draw the Reviewer’s attention to the scale of the x-axis. In some cases, the width of the CIs is augmented by the small scale of the x-axis (e.g., cognitive and socio-emotional development). In other cases, the CIs are indeed wide. We have now commented on the width of the CIs and the trends in the Discussion section (lines 440-445): *“Overall, most associations were small with relatively wide confidence intervals, indicating that moderate to very small associations were possible. Surprisingly, associations between women’s empowerment and child development were not monotonic, i.e., the magnitude of the associations was similar across quintiles of women’s empowerment and its dimensions. In contrast, associations between women’s empowerment and parental stimulation generally increased in higher empowerment quintiles.”*

**13.     With all due respect to the authors, I cannot agree with the first statement in the discussion. The results show many more null associations than positive associations, and where there is some association, they are quite weak and small. This is more likely due to the indicators used than the lack of effect of women's empowerment.**

Thank you for raising these concerns. We have revised the paragraph, used more cautionary language, and highlighted that our results are suggestive and, in some cases, quite limited (lines 429-445): *“In this study, we found evidence suggesting that women’s empowerment was positively associated with child cognitive development, growth, early learning, and nutrition outcomes among children 36-59 months of age in SSA. However, with respect to child growth and nutrition, evidence was limited with associations significant only among women in the highest empowerment quintile, relative to the lowest. We found no evidence that women’s empowerment or its dimensions were associated with child literacy-numeracy development. With respect to socio-emotional and physical development, we only observed associations with specific empowerment dimensions. Different dimensions of women’s empowerment were associated with different outcomes: higher access to and control over resources was predictive of better socio-emotional development, whereas higher decision-making power was predictive of better cognitive and physical development. Women’s empowerment and all its dimensions were predictive of learning resources and parental stimulation. Overall, most associations were small with relatively wide confidence intervals, indicating that moderate to very small associations were possible. Surprisingly, associations between women’s empowerment and child development were not monotonic, i.e., the magnitude of the associations was similar across quintiles of women’s empowerment and its dimensions. In contrast, associations between women’s empowerment and parental stimulation generally increased in higher empowerment quintiles.”*

**14.     Again, in line 368, the assertion needs to be corrected.**

[Please see our response to comment #2]
