# Supplementary material for: Associations between women’s empowerment and child development, growth, and nurturing care practices in sub-Saharan Africa: A cross-sectional analysis of demographic and health survey data
Source: PLoS Med. 2021 Sep 16;18(9):e1003781. doi: 10.1371/journal.pmed.1003781 (PMC8483356; doi:10.1371/journal.pmed.1003781)
Supplement: Attachment — Submitted filename: R3 Responses to Comments.docx [file pmed.1003781.s015.docx]

We thank the Reviewers and the Editors for their time and thoughtful suggestions for improving our paper. We have provided point-by-point responses to their concerns and comments below.

**EDITORS**

**In the submission form, you mention that restrictions exist on data access, but are these data not freely available from DHS? Please amend the statement either to explain briefly what the restrictions are, or alter it to "Yes - all data freely available" or similar.**

We have altered the Data Availability statement to “Yes – all data are freely available” and have amended the description of where the data can be found to: *“The data underlying the results presented in the study are publicly available from the DHS Program (http://www.dhsprogram.com). Registration is required to access the data.”*

**Please remove the information on funding, competing interests and data access from the title page. This information will appear in the article metadata in the event of publication, via entries in the submission form.**

We have removed this information from the title page.

**Acknowledgements should appear at the end of the main text.**

We have moved the Acknowledgements to the end of the main text.

**Please list the country names in the abstract.**

We have listed the nine countries in Methods and Findings section of the Abstract (lines 11-12)

**Please quote aggregate demographic details for study participants in the abstract.**

We have briefly described demographics of the analytic sample in the Methods and Findings section of the Abstract (lines 20-22): *“On average, households in our sample were large (8.5±5.7 members) and primarily living in rural areas (71%). Women were 31±6.6 years on average, 54% had no education, and 31% had completed primary education.* *Children were 47±7 months old and 49% were female.”*

**Please correct the typo in reference 70.**

Thank you for catching this typo. We have reviewed all references again to make sure there are no additional typos.

-----------------------------------------------------------

**REVIEWER 1**

**This paper reports an investigation of empowerment and child development using a secondary data analysis. I was asked for a statistical report and I interpret that to include all aspects of the design and conduct of the study. When I am asked to look at a re-submission which I had already reviewed on a previous submission I restrict myself on principle to commenting on how the authors have responded to my original comments. I do not review the article again from scratch unless it has been so changed as to amount effectively to a new submission.**

Thank you for your time and suggestions for improving our paper.

**I said:**

***Naming factors is always a tricky issue but given the content of the items which go to make up the authors' gender dimension why was it not named `intimate partner violence'? I am not an expert in this area but is there not more to gender roles than physical assault? Pages 22 and 23 seem to agree. Obviously if that is all DSS asks about then nothing can be done by the authors to broaden the scope of the factor analysis.***

**The authors attempt to justify this in the rebuttal but really their statement there seems to amount to saying that they would have liked to have a factor embracing all the concepts they mention but they have not been able to have one because of limitations in the data but they still intend to call it Gender. I think it would be better to report what was actually found not what was desired to be found.**

Thank you for reiterating this point. We have renamed the factor score to “Attitudes towards wife beating” to more accurately reflect the indicators making up the factor score. We have retained “Gender” only in the section laying out the Conceptual Framework, which is broader and encompasses theoretical and empirical literature beyond DHS data. We note on lines 237-238 why we have renamed this dimension: *“Since the “Gender” dimension only contained indicators on attitudes towards wife beating, we renamed this dimension to “Attitudes towards wife beating”.* Subsequently, we refer to the dimension as “Attitudes toward wife beating” and have made relevant changes throughout the manuscript and supplements. For brevity, we refer the Reviewer to the text.

**I said:**

***There are only four quintiles. if `fifth' lacks academic credentials then `quintile category' might be better.***

**The authors mis-understand my point. There is one median which divides the sample into halves, two tertiles which divide the sample into thirds, three quartiles which divide the sample into quarters, and four quintiles which divide the sample into fifths. There is the potential for ambiguity here as quintile categories could be used in modelling as could the actual quintiles either as a categorical predictor or as a continuous one.**

Thank you for the clarification. We have revised and use “quintile category” throughout. For brevity, please see the main text and supplemental materials.

**I said:**

***This tells us about missing data on confounders but what about missing data on the empowerment items?***

**The authors state "Missing data on any of the exposure indicators or outcomes was not imputed to reduce the risk of bias." It is complete cases analysis which introduces potential bias not multiple imputation. The authors' comment here does not justify ignoring the missingness.**

Thank you for reiterating this concern. We agree with the Reviewer that complete case analysis may introduce bias in certain situations and have clarified in the manuscript why we believe our use of complete case analysis is justified (lines 319-330): *“Missing data on any of the confounders was imputed using mean imputation (N=6 observations with missing data on household head’s age). Missing data on any of the exposure indicators were handled through the use of a full weight matrix by the CFA model estimator. The proportions of missing data on child development, growth, and early learning outcomes were 4.3%, 4.9%, and 3.2%, respectively, below the 5% threshold typically recommended for complete case analysis [54]. Child diet was collected for a randomly selected sub-sample of children and was therefore missing for 47% of children by design. Thus, only outcome variables had missing values and none of the exposure or confounder variables used in the models* *had missing values. Therefore, we used complete case analysis since in this case multiple imputation does not provide any addition information, yields similar estimates if the same predictors of missingness are used, and may in fact introduce uncertainty and increase standard errors [54–56].”*

New references:

54.	Jakobsen JC, Gluud C, Wetterslev J, Winkel P. When and how should multiple imputation be used for handling missing data in randomised clinical trials – a practical guide with flowcharts. BMC Med Res Methodol. 2017;17: 162. doi:10.1186/s12874-017-0442-1

55.	Garson GD. Missing Values Analysis and Data Imputation. Asheboro, NC: Statistical Associates Publishers; 2015.

56.	Groenwold RHH, Donders ART, Roes KCB, Harrell FE, Moons KGM. Dealing With Missing Outcome Data in Randomized Trials and Observational Studies. Am J Epidemiol. 2012;175: 210–217. doi:10.1093/aje/kwr302

**The authors' discussion starts**

**In this study, we found evidence suggesting that women's empowerment was positively associated with child cognitive development, growth, early learning, and nutrition outcomes among children 36-59 months of age in SSA.**

**I still feel that even in its toned down form that discussion over-interprets Table 3 and 4. Unless there was a prior hypothesis that just those categories would differ from the lowest on some but not all outcome variables then, in the absence of an overall test of difference, this is very data-driven.**

Thank you for reiterating this point. We have revised the first sentence of the Discussion to further tone-down the language and clarify that the evidence we found is limited and suggestive for some outcomes but more consistent for other outcomes (lines 448-451): *“In this study, we found limited evidence suggesting that women’s empowerment may be positively associated with child cognitive development, growth, and nutrition outcomes among children 36-59 months of age in SSA, and more consistent evidence that women’s empowerment and all its dimensions were predictive of learning resources and parental stimulation.”*
